# A Corrosion-Layered Multi-Scale Homogenization Framework for Creep Prediction of Sulfate-Attacked Concrete

**DOI:** 10.3390/ma19214586

**Published:** 2026-10-29

**Authors:** Yang Yu, Lijia Wang, Jinlong Liu, Kunyang Yu, Junqi Lin

**Affiliations:** 1 Tangshan Open University, Tangshan 063000, China; yunnysummer@163.com; 2 Key Laboratory of Earthquake Engineering and Engineering Vibration, Institute of Engineering Mechanics, China Earthquake Administration, Harbin 150080, China; liujinlong@iem.ac.cn (J.L.); linjunqi1964@163.com (J.L.); 3 Key Laboratory of Earthquake Disaster Mitigation, Ministry of Emergency Management, Harbin 150080, China; 4 College of Hydraulic Science and Engineering, Northeast Agricultural University, Harbin 150030, China; yukunyang@neau.edu.cn

**Keywords:** concrete, sulfate attack, creep, multi-scale homogenization, interfacial transition zone

## Abstract

**Highlights:**

1. A corrosion-layered multiscale model is proposed for sulfate-attacked concrete creep.
2. ITZ corrosion degree decays exponentially with depth.
3. Mori–Tanaka homogenization is coupled with a Burgers creep model.
4. The model reproduces modulus evolution and predicts creep compliance.
5. Sensitivity analyses identify dominant long-term creep parameters.

**Abstract:**

Sulfate attack progressively degrades concrete stiffness and accelerates long-term creep. Existing creep models mainly address sound concrete and do not represent the spatial gradient of corrosion products within the interfacial transition zone (ITZ). This study develops a multi-scale homogenization framework, coupling phase evolution, layered ITZ corrosion, and viscoelastic creep. The ITZ is discretized into sub-layers, with corrosion degree decaying exponentially from the aggregate surface, and the decay coefficient is calibrated from measured sulfate concentration profiles. Phase volume fractions are updated with corrosion degree, and the effective elastic modulus is obtained using Mori–Tanaka homogenization. Creep compliance is then predicted with a Burgers model, whose parameters depend on the homogenized modulus and corrosion product content. The framework reproduces the non-monotonic evolution of elastic modulus under different sulfate concentrations, with a mean absolute percentage error below 10%. At a corrosion degree of 0.3, the predicted relative creep increment reaches approximately 11% at 365 days. The effects of temperature, loading age, and wet–dry cycles are also quantified. Sensitivity analyses identify the long-term creep modulus and corrosion product coefficient as the dominant parameters. The model provides a physically grounded tool for assessing the long-term deformation of sulfate-attacked concrete.

## 1. Introduction

Sulfate attack is a major durability problem for concrete structures in marine environments and sulfate-rich soils [1,2,3,4,5]. Sulfate ions penetrating into the pore system react with cement hydrates and form expansive products such as ettringite and gypsum [3,4,5,6,7]. These products initially fill pores and increase stiffness, but progressive crystallization pressure leads to microcracking and strength loss [8,9,10,11]. The elastic modulus of concrete first increases and then decreases during sulfate exposure, reflecting a competition between pore filling and damage [12,13,14,15,16]. Creep deformation is equally important because it affects long-term serviceability and stress redistribution [17,18,19,20,21,22,23].

Creep of concrete has been studied through various approaches [17,18,20,24,25,26,27,28,29,30,31,32]. Empirical models describe the time-dependent deformation of sound concrete under sustained load [33]. These models are widely used in design codes but are calibrated for normal conditions and do not account for chemical degradation. Rheological models represent creep using combinations of springs and dashpots, such as the Burgers model and the Kelvin chain model [19,22,33]. These models can capture the main features of creep compliance and are convenient for numerical implementation. Micromechanical models link creep to the properties of individual phases, typically using homogenization methods. In this approach, the creep of C-S-H gel is upscaled to cement paste, mortar, and concrete through analytical or numerical schemes [19,20,22,25,27,29,31,34]. Multi-scale homogenization methods have proven successful for elastic properties of cementitious materials [3,19,20,22,35,36,37,38,39,40,41]. They represent concrete as a hierarchical composite, spanning from C-S-H gel to concrete, and analytical schemes such as the Mori–Tanaka and self-consistent methods allow upscaling from individual phases to the macroscopic scale [42,43,44,45,46,47,48]. Recent extensions include the incorporation of the ITZ as a distinct phase, as well as the updating of phase volume fractions according to corrosion reactions [3,5,49,50]. However, most existing creep models are developed for sound concrete and do not account for chemical degradation [17,28,31,32,51]. The influence of sulfate attack on creep remains poorly understood [52,53], despite its relevance for structures in aggressive environments. Existing corrosion models typically treat the ITZ as uniformly corroded [3,5], ignoring the spatial gradient of sulfate concentration. In reality, sulfate concentration decreases with the depth from the aggregate surface, causing a corresponding gradient between corrosion degree and mechanical degradation. Neglecting this gradient can lead to inaccurate predictions of both stiffness and creep. Therefore, a predictive framework that links microstructural changes caused by sulfate attack to macroscopic elastic and creep properties is urgently needed.

The present study addresses the lack of creep models that can reflect the spatial gradient of sulfate-induced damage in concrete. Existing creep models rely on empirical or rheological parameters that are either determined for sound concrete or applied uniformly across the entire ITZ. These models cannot capture the experimentally observed fact that sulfate concentration and corrosion damage decrease with the depth from the aggregate surface. As a result, they are unable to explain how local differences in phase composition and stiffness within the ITZ translate into the macroscopic creep behavior of sulfate-attacked concrete. In the proposed framework, the multi-scale homogenization method is coupled with a Burgers creep model in a way that removes this limitation. The ITZ is divided into sub-layers, and each sub-layer is assigned a local corrosion degree that decays exponentially from the aggregate surface. The phase volume fractions in each sub-layer are then updated according to the local corrosion degree. The instantaneous elastic modulus used in the Burgers model is obtained from Mori–Tanaka homogenization of these locally varying phases, and the creep parameters are modified by the corrosion product content. This integration ensures that both the elastic and creep responses are derived from the same spatially resolved microstructure. The model therefore provides a unified route from the local corrosion state to the long-term deformation of concrete, which is a capability not offered by previous uniform corrosion models. By explicitly linking the spatial gradient of corrosion to the creep parameters, the framework resolves a key gap in existing creep prediction methods for sulfate-attacked concrete. It can account for the dependence of creep compliance on the corrosion degree, the ITZ decay coefficient, and the volume fraction of corrosion products. This enables the model to capture not only the overall acceleration of creep with corrosion but also the influence of the corrosion profile shape on long-term deformation. Such information is essential for assessing the serviceability of concrete structures in marine and sulfate-rich environments, where the corrosion-affected layer thickness is often a non-negligible fraction of the structural dimensions.

The remainder of this paper is organized as follows. Section 2 describes the multi-scale homogenization method and the phase evolution model. Section 3 presents the creep model and its integration with the homogenization framework. Section 4 compares model predictions with experimental data and discusses sensitivity results. Section 5 summarizes the main findings and conclusions.

## 2. Multi-Scale Homogenization with Corrosion Layering

**Figure 1:**
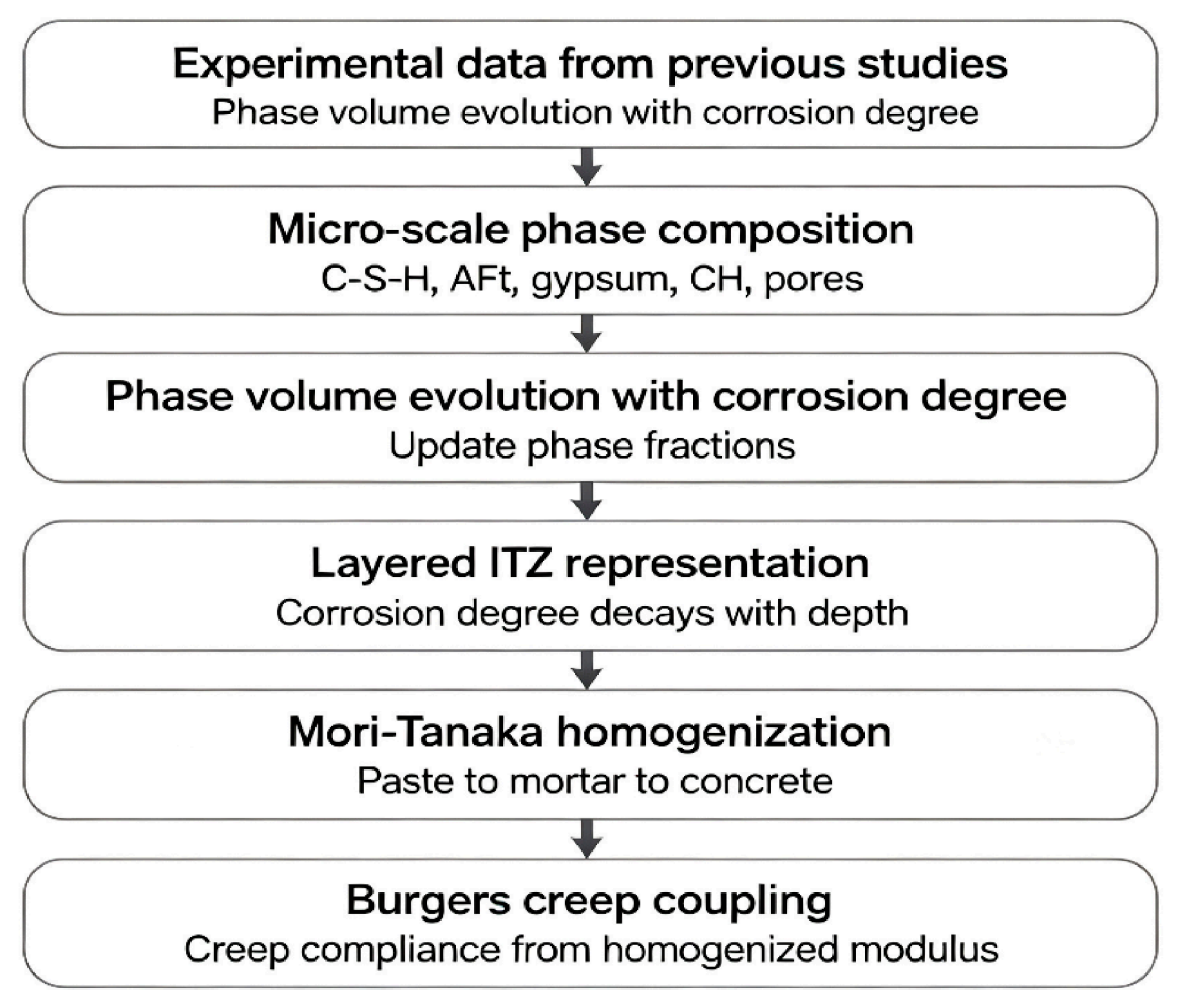
Overall research framework of this study.

The overall research framework of this study is illustrated in Figure 1. The framework begins with the experimental data obtained from previous studies, which include the phase volume fractions, the elastic modulus, and the sulfate concentration profiles. These data are used to establish the relationships between the corrosion degree and the volume fractions of major phases. The framework then proceeds to the micro-scale phase composition, where the hydration and corrosion products are represented as distinct phases with their own elastic and creep properties. On this basis, the phase volume evolution corresponding to the corrosion degree is introduced, and the volume fractions of AFt, gypsum, CH, C-S-H, and pores are updated for each corrosion state. The layered ITZ representation is then constructed, in which the ITZ is divided into sub-layers, and the corrosion degree is assumed to decay exponentially with depth. After that, Mori–Tanaka homogenization is applied to upscale the elastic modulus from the cement paste scale to the mortar scale and further, to the concrete scale. Finally, the homogenized elastic modulus is coupled with a Burgers creep model, and the creep compliance is computed, taking the corrosion product content into account. The framework is subsequently validated against independent experimental data, and sensitivity analyses are performed to quantify the influence of the key parameters.

### 2.1. Multi-Scale Representation and Phase Properties

**Figure 2:**
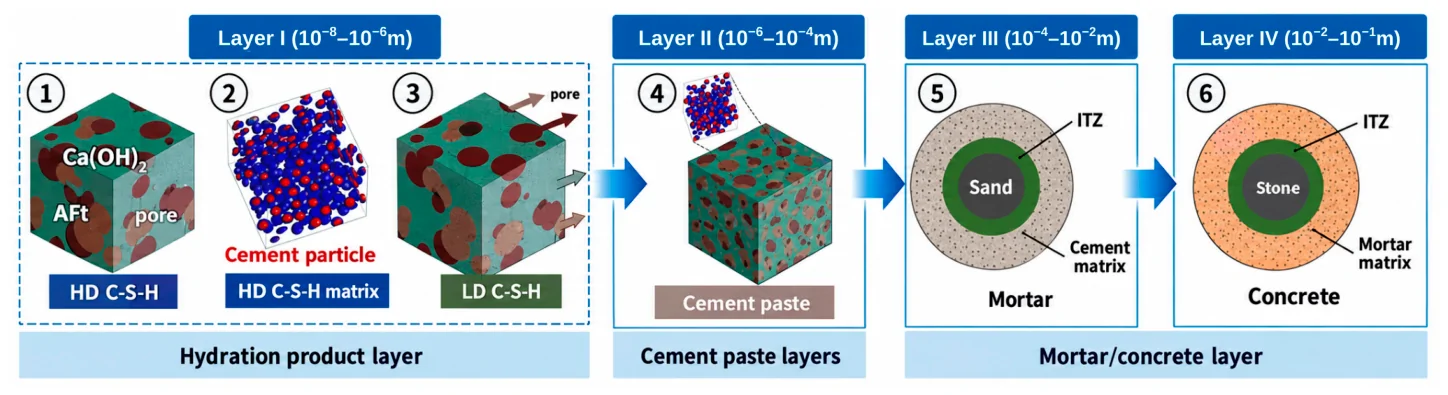
Multi-scale homogenization framework of concrete.

The adopted multi-scale representation follows the four-level scheme of [3,5], as shown in Figure 2. Level I represents hydration products, including high-density C-S-H (HD C-S-H), low-density C-S-H (LD C-S-H), portlandite (CH), ettringite (AFt), gypsum, capillary pores, and unhydrated clinker. Level II corresponds to cement paste, where the ITZ layer surrounds the inner hydration products. Level III describes mortar as a cement paste matrix with fine aggregates coated by the ITZ. Level IV represents concrete as a mortar matrix with coarse aggregates coated by the ITZ. The present study follows this same four-level structure and applies it to both elastic and creep calculations. The elastic properties of individual phases are listed in Table 1. In addition to elastic constants, the creep behavior of the cementitious phases requires viscoelastic parameters. The contact creep modulus and characteristic time for LD C-S-H and HD C-S-H are adopted from Vandamme and Ulm [23] and are given in Table 2. The corrosion products AFt and gypsum are assumed to have the same creep parameters as LD C-S-H, in the absence of direct experimental data. Their influence is further examined through sensitivity analysis.

**Table 1:** Elastic properties and aspect ratios of individual phases [19,22,54].

Phase	Elastic Modulus (GPa)	Poisson Ratio	Aspect Ratio
HD C-S-H	29.4	0.24	1.0
LD C-S-H	21.7	0.24	1.0
Clinker	140.0	0.30	1.0
CH	38.0	0.305	0.1
AFt	42.3	0.324	10.0
Gypsum	45.7	0.33	0.1
Sand	50.0	0.20	1.0
Stone	50.0	0.20	1.0

**Table 2:** Creep parameters of C-S-H phases [23].

Phase	Contact Creep Modulus (GPa)	Characteristic Time (s)
LD C-S-H	112.2	1.63
HD C-S-H	182.5	1.63

The complete mix proportions and raw material characteristics of the validation concrete are summarized in Table 3. The oxide composition is taken from Cheng et al. [12]. The mineralogical composition is calculated using the Bogue equations from this oxide composition; no quantitative XRD/Rietveld analysis was reported in the original study. The model is developed for OPC-based concrete with siliceous aggregates and comparable aluminate contents. Extension to other cement types or reactive aggregates requires recalibration.

The aggregates are assumed to be chemically inert and linearly elastic under sulfate attack. This assumption is reasonable for the siliceous aggregates used in the referenced studies. The sensitivity of the predicted creep compliance to the ITZ thickness is analyzed in Section 4.4.3. The creep compliance is more sensitive to the ITZ thickness than to the aggregate modulus, which is consistent with the ITZ being the weakest region in concrete. For reactive aggregates, such as limestone or recycled aggregates, the chemical interaction between the aggregate and the sulfate solution may alter the ITZ properties. In such cases, the model requires recalibration of the ITZ phase–evolution relationships. This limitation is discussed in Section 4.5.

### 2.2. Phase Volume Evolution Under Sulfate Attack

The phase volume fractions are derived from the experimental measurements reported by Jia et al. [3,5]. In that study, cement paste specimens were exposed to 15% Na_2_SO_4_ solution for durations up to 400 days, and the volume fractions of the hydration and corrosion products were quantified using X-ray diffraction and thermogravimetric analysis. The measured data were used to establish relationships between the corrosion degree (CD) and the phase volume fractions. These relationships, which are adopted in the present study, are given by Equations (1)–(6). The CD is defined as the volume fraction of chemically altered cement paste. The phase volume fractions in the ITZ and the bulk paste are functions of CD.

Based on the experimental data reported by Jia et al. [3,5], the following linear relationships are adopted for the ITZ phase composition.

The volume fraction of AFt is given by

(1) $$f_{\mathrm{AFt}}=f_{\mathrm{AFt}}^{0}+0.30\mathrm{CD}$$

The volume fraction of gypsum is given by

*f*_gypsum_ = 0.25*CD* (2)

The volume fraction of CH is given by

(3) $$f_{\mathrm{CH}}=f_{\mathrm{CH}}^{0}-0.20\mathrm{CD}$$

The volume fraction of LD C-S-H is given by

(4) $$f_{\mathrm{LD}}=f_{\mathrm{LD}}^{0}-0.30\mathrm{CD}$$

The volume fraction of HD C-S-H is given by

(5) $$f_{\mathrm{HD}}=f_{\mathrm{HD}}^{0}-0.05\mathrm{CD}$$

The volume fraction of pore is given by

(6) $$f_{\mathrm{pore}}=f_{\mathrm{pore}}^{0}-0.10\mathrm{CD}+0.15CD^{2}$$

where the superscript 0 denotes the initial volume fraction in the uncorroded state. The initial volume fractions for the ITZ are $f_{\mathrm{AFt}}^{0}=0.09$, $f_{\mathrm{CH}}^{0}=0.132$, $f_{\mathrm{LD}}^{0}=0.419$, $f_{\mathrm{HD}}^{0}=0.274$, and $f_{\mathrm{pore}}^{0}=0.36$. The phase–evolution relationships are given by Equations (1)–(6). The initial volume fractions for the ITZ are $f_{\mathrm{AFt}}^{0}=0.09$, $f_{\mathrm{CH}}^{0}=0.132$, $f_{\mathrm{LD}}^{0}=0.419$, $f_{\mathrm{HD}}^{0}=0.274$, and $f_{\mathrm{pore}}^{0}=0.36$. These values sum to 1.275 and are first normalized by dividing by 1.275, giving $f_{\mathrm{AFt}}^{0}=0.0706$, $f_{\mathrm{CH}}^{0}=0.1035$, $f_{\mathrm{LD}}^{0}=0.3286$, $f_{\mathrm{HD}}^{0}=0.2149$, and $f_{\mathrm{pore}}^{0}=0.2824$. Equations (1)–(6) are then applied to these normalized initial values, and the resulting fractions are normalized again to sum to unity. Table 4 presents the mass/volume balance over CD = 0–0.5. The coefficients in Equations (1)–(6) are phenomenological fitting parameters calibrated from the experimental data of Jia et al. [3,5]; after normalization, they do not represent strict chemical stoichiometry. The normalization factor varies between 1.0000 and 1.0168 over CD = 0–0.5, so the physical meaning of the individual coefficients is preserved within 1.7%. The linear term in Equation (6) accounts for pore filling by ettringite and gypsum, while the quadratic term accounts for microcracking at high corrosion degrees.

The model is calibrated and validated over the CD range 0–0.5. Beyond CD = 0.5, the quadratic term in Equation (6) begins to dominate, and the coefficients may no longer represent the physical processes accurately. This limitation is stated explicitly in Section 4.5.

The exposure conditions of the referenced studies differ in several respects. Gong et al. [52] tested OPC-based concrete under 1%, 5%, and 10% Na_2_SO_4_ solutions for up to 540 days under continuous immersion and sustained load. Cheng et al. [12] tested OPC concrete under 3%, 5%, and 10% Na_2_SO_4_ solutions, with a wet–dry regime of 7 days immersion followed by 8 days drying, for up to 360 days, with monthly renewal of the solution. Piasta and Schneider [55] tested OPC concrete under 1% and 5% Na_2_SO_4_ solutions for up to 1140 days under continuous immersion and sustained compression. The phase–evolution relationships determined for cement paste exposed to 15% Na_2_SO_4_ [3,5] are transferred to concrete through the local corrosion degree CD. This transfer assumes that the local phase assemblage depends primarily on CD and is valid for OPC-based systems with comparable cement chemistry. The differences in exposure conditions (concentration, wet–dry regime, duration) are reflected in the value of CD, which is mapped from the environmental exposure through the sulfate concentration profile and the decay coefficient β. For different cements, sulfate concentrations, pH, or exposure regimes that fall outside the range covered by these studies, recalibration is required.

### 2.3. Layered ITZ and Spatial Corrosion Degree

**Figure 3:**
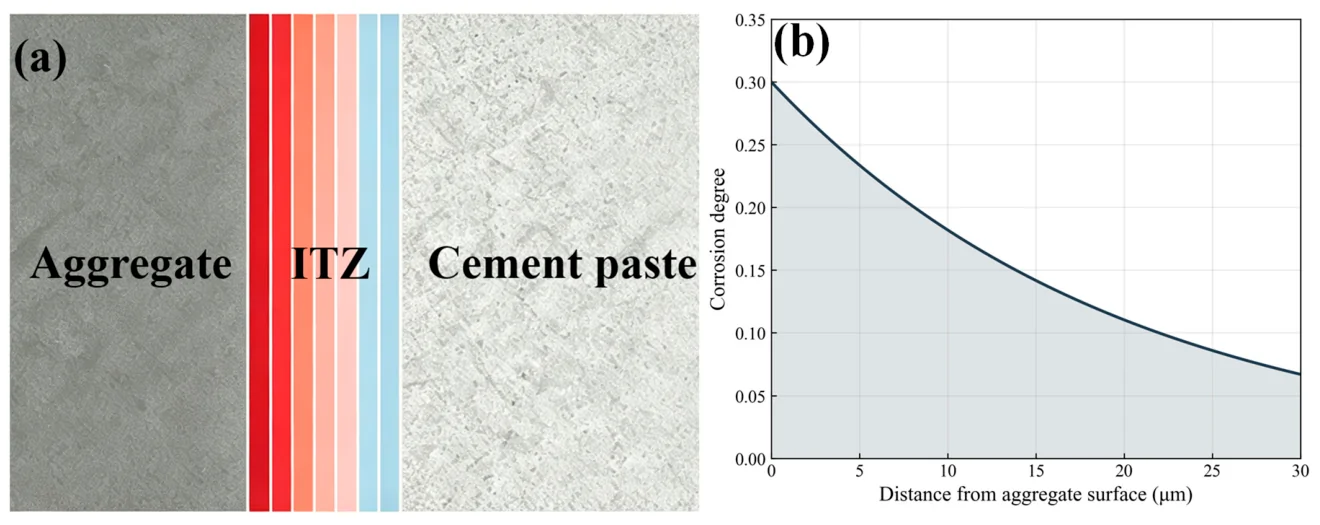
(**a**) Layered representation of the ITZ and (**b**) exponential decay of corrosion degree across the ITZ thickness.

The spatial distribution of corrosion degree across the ITZ is not uniform because sulfate concentration decreases with depth from the aggregate surface. This gradient is quantified by an exponential decay function:

*CD*(*x*) = *CD_surface_* exp(−*βx*) (7)

where CD_surface_ is the CD the aggregate surface, and *β* is a decay coefficient. The decay coefficient is calibrated from sulfate concentration profiles obtained by inductively coupled plasma spectroscopy on concrete specimens. The decay coefficient *β* is calibrated from the sulfate concentration profiles reported by Cheng et al. [12], which were obtained by chemical titration of ${\mathrm{SO}}_{4}^{2-}$ in concrete slices at depths from 0 to 20 mm. Fitting the measured distributions to *C*(*x*) = *C*_0_exp(−*βx*), where *C*(*x*) is the sulfate concentration at depth *x*, and *C*_0_ is the surface concentration, gives *β* ≈ 0.30 mm^−1^. With *h*_ITZ_ = 0.03 mm, the product *β* ⋅ *h*_ITZ_ = 0.009≪1, indicating that the corrosion degree is essentially uniform within the ITZ thickness under the exposure conditions considered here. The average corrosion degree of the ITZ, used when a layered calculation is not performed, is given by

(8) $$\bar{\mathrm{CD}}=\frac{1}{h_{\mathrm{ITZ}}}\int_{0}^{h_{\mathrm{ITZ}}}CD_{\mathrm{surface}}\exp(-\mathrm{\beta x})\mathrm{dx}=\frac{CD_{\mathrm{surface}}}{\beta h_{\mathrm{ITZ}}}\left[1-\exp(-\beta h_{\mathrm{ITZ}})\right]$$

where *h*_ITZ_ is the ITZ thickness, taken as 30 μm. For the layered calculations, the ITZ is divided into *n*_layers_ sub-layers of equal thickness, and the corrosion degree at the center of each sub-layer is computed from Equation (7). The phase volume fractions in each sub-layer are then obtained using Equations (1)–(6) with the local CD value. This layered representation allows the elastic and creep properties to vary continuously across the ITZ, capturing the transition from severely damaged outer regions to relatively intact inner regions. Figure 3a presents the layered representation of the ITZ. The zone is divided into several thin sub-layers between the aggregate surface and the bulk cement paste. The color of the sub-layers changes gradually from dark red near the aggregate to light blue near the cement paste, indicating a decreasing corrosion degree with depth. Figure 3b shows the corresponding exponential decay of the corrosion degree as a function of the distance from the aggregate surface. The corrosion degree decreases from the surface value to a lower value within the thickness of the ITZ. This spatial distribution is used to assign different phase volume fractions and elastic properties to each sub-layer in the homogenization procedure.

### 2.4. Mori–Tanaka Homogenization

The effective elastic properties at each scale are obtained using the Mori–Tanaka scheme [56]. This method is widely used for composites with spherical inclusions and provides explicit expressions for the effective bulk and shear moduli. The bulk modulus *K* and shear modulus *G* of a composite with matrix moduli of *K*_0_ and *G*_0_ and inclusions of moduli *K_i_* and *G_i_* at a volume fraction *f_i_* are updated sequentially. The effective moduli after adding inclusion phase *i* are

*K*_eff_ = *K*_0_ + *f_i_*(*K_i_* − *K*_0_)*α_K_* (9)

*G*_eff_ = *G*_0_ + *f_i_*(*G_i_* − *G*_0_)*α_G_* (10)

where *α_K_* and *α_G_* are the dilute strain concentration factors for spherical inclusions, given by

(11) $$\alpha_{K}=\frac{K_{0}+\frac{4}{3}G_{0}}{K_{i}+\frac{4}{3}G_{0}}$$

(12) $$\alpha_{G}=\frac{G_{0}+F_{0}}{G_{i}+F_{0}}$$

where *F*_0_ is given by

(13) $$F_{0}=\frac{G_{0}}{6}\frac{9K_{0}+8G_{0}}{K_{0}+2G_{0}}$$

where *K*_eff_ and *G*_eff_ are the effective bulk and shear moduli, *K*_0_ and *G*_0_ are the matrix moduli, *K_i_* and *G_i_* are the inclusion moduli, and *f_i_* is the inclusion volume fraction. The pore phase is treated as an inclusion with a very small modulus to avoid numerical singularity. The volume fractions of sand and coarse aggregate are taken as 0.231 and 0.458, respectively, based on the mix proportions.

## 3. Creep Model and Coupling with Multi-Scale Homogenization

This study presents the creep model and its integration with the multi-scale homogenization framework developed in the preceding chapter. The Burgers viscoelastic model is first introduced, and its parameters are defined. The influence of corrosion products on the Kelvin modulus and Maxwell viscosity is then described. Finally, the sequential homogenization procedure that links the instantaneous elastic modulus from the Mori–Tanaka scheme to the Burgers model is explained, with particular attention to the layered ITZ representation and the upscaling from cement paste to concrete.

### 3.1. Burgers Creep Model

The creep compliance of concrete is described by a Burgers model, which is a classical viscoelastic model consisting of a Maxwell unit and a Kelvin unit, in series [21]. The compliance function is given by

(14) $$J(t)=\frac{1}{E_{M}}+\frac{t}{\eta_{M}}+\frac{1}{E_{K}}\left[1-\exp\left(-\frac{t}{\tau_{K}}\right)\right]$$

where *E_M_* is the instantaneous elastic modulus, *η_M_* is the Maxwell viscosity, *E_K_* is the Kelvin spring modulus, and *τ_K_* is the Kelvin characteristic time. The first term represents instantaneous elastic deformation, the second term represents viscous flow, and the third term represents delayed elastic deformation.

In the present multi-scale framework, the *E_M_* is not an independent parameter but is obtained from the Mori–Tanaka homogenization described in Section 2. The long-term creep modulus *E_c_* is taken from Binder et al. [17] as 41.6 GPa at 20 °C. The Maxwell viscosity is defined as

*η_M_* = *E_c_t*_char_ (15)

where *t*_char_ is a characteristic time controlling the steady-state creep rate, set to 500 days in this study. The Kelvin characteristic time *τ_K_* is set to 10 days, which is consistent with the typical time scale of short-term creep in concrete.

The long-term compliance of the Burgers model approaches the asymptotic value:

(16) $$J_{\infty }=\frac{1}{E_{M}}+\frac{1}{E_{K}}$$

where *J*_∞_ is the compliance after the Kelvin unit has fully relaxed but before the viscous flow dominates. The creep rate at large times is governed by the Maxwell dashpot and is expressed as

(17) $${\dot{J}}_{\infty }=\frac{1}{\eta_{M}}$$

Equation (17) shows that the steady-state creep rate is inversely proportional to the Maxwell viscosity. In the present framework, both *E_M_* and *E_K_* depend on the corrosion state, so the long-term compliance also changes with the CD.

### 3.2. Corrosion Effect on Creep Parameters

The effect of corrosion is incorporated through the volume fraction of the corrosion product *f*_corr_ = *f*_AFt_ + *f*_gypsum_. Experimental observations indicate that corrosion products have higher stiffness than that of C-S-H, but their formation is accompanied by microcracking, leading to a complex influence on creep [3,50,53]. In this model, the Kelvin modulus and Maxwell viscosity are modified as

*E_K_* = *E_c_*scale(1 + 1.2*f*_corr_) (18)

*η_M_* = *E_c_t*_char_scale(1 − 0.6*f*_corr_) (19)

where the scale factor is the ratio of the current elastic modulus to the uncorroded modulus. The term (1 + 1.2*f*_corr_) accounts for the stiffening effect of corrosion products on the short-term creep. The term (1 − 0.6*f*_corr_) accounts for the acceleration of long-term creep due to damage. These expressions are empirical and are calibrated using the experimental creep data of Gong et al. [52].

The corrosion product volume fraction is defined as

*f*_corr_ = *f*_AFt_ + *f*_gypsum_ (20)

where *f*_AFt_ and *f*_gypsum_ are obtained from Equations (1) and (2). This fraction represents the total volume of the expansive products formed during sulfate attack. It is used to modify the creep parameters because these products have different stiffness and damage characteristics compared with those of C-S-H. To quantify the stiffness loss caused by corrosion, a relative stiffness degradation factor *s_E_* is introduced as

(21) $$s_{E}=\frac{E_{M}}{E_{M0}}$$

where *E_M_*_0_ is the instantaneous elastic modulus of the uncorroded concrete. The stiffness degradation factor increases from zero in the sound state to positive values as corrosion progresses.

Substituting Equation (21) into Equations (18) and (19) gives the explicit dependence of the creep parameters on damage, i.e.,

*E_K_* = *E_c_s_E_*(1 + 1.2*f*_corr_) (22)

*η_M_* = *E_c_t*_char_*s_E_*(1 − 0.6*f*_corr_) (23)

The factor *s_E_* is a phenomenological stiffness degradation factor, not a stiffness degradation factor in the strict sense of continuum damage mechanics. It does not have an independent evolution law or an irreversibility condition. This simplification is sufficient for the present homogenization framework, in which stiffness loss is obtained directly from the Mori–Tanaka homogenization.

### 3.3. Multi-Scale Integration of Creep

The multi-scale creep model is implemented in a sequential manner. At each time step, the local corrosion degree in each ITZ sub-layer is determined from Equation (7). The phase volume fractions are then computed from Equations (1)–(6). The elastic modulus of each sub-layer is obtained by the Mori–Tanaka scheme. The instantaneous elastic modulus of concrete is then upscaled from paste to mortar and concrete. This modulus is inserted into the Burgers model as *E_M_*. The corrosion product volume fraction used in Equations (18) and (19) is calculated as the volume-weighted average over the ITZ and the bulk paste. The resulting compliance function *J*(*t*) describes the creep deformation of the homogenized concrete.

The instantaneous elastic modulus of concrete is obtained from the homogenized bulk and shear moduli as

(24) $$E_{M}=\frac{9K_{\mathrm{conc}}G_{\mathrm{conc}}}{3K_{\mathrm{conc}}+G_{\mathrm{conc}}}$$

where *K*_conc_ and *G*_conc_ are the effective bulk and shear moduli of concrete at the current corrosion state. The volume fraction of the corrosion product used in Equations (18) and (19) is computed as the volume-weighted average over the ITZ sub-layers and the bulk paste. For the layered ITZ, this average is expressed as

(25) $${\bar{f}}_{\mathrm{corr}}=\frac{1}{h_{\mathrm{ITZ}}}\sum_{i=1}^{n_{\mathrm{layers}}}f_{\mathrm{corr},i}\Delta x_{i}$$

where Δ*x_i_* is the thickness of the *i*-th sub-layer, and *f*_corr,*i*_ is the corrosion product volume fraction in that sub-layer. When a uniform ITZ is assumed, ${\bar{f}}_{\mathrm{corr}}$ reduces to the value at the average corrosion degree.

To clarify how the multi-scale homogenization influences the creep parameters, the following steps are performed. First, the elastic modulus of the cement paste is computed with the Mori–Tanaka scheme using the phase volume fractions determined by the local corrosion degree. Second, the elastic modulus of mortar is obtained by adding fine aggregates to the cement paste. Third, the elastic modulus of concrete is obtained by adding coarse aggregates to the mortar. The resulting concrete elastic modulus is *E_M_*. This procedure ensures that the corrosion-induced changes in microstructure are consistently reflected in the creep model. For a layered ITZ treatment, the ITZ is divided into sub-layers. Each sub-layer has its own corrosion degree and thus, its own phase composition and elastic modulus. The effective ITZ modulus is computed as the volume-weighted average of the sub-layer moduli. This effective modulus is then used in the homogenization of the cement paste. This approach captures the gradual variation in stiffness across the ITZ thickness. In the uncorroded state, the corrosion degree is zero, so the phase volume fractions take their initial values, and the stiffness degradation factor in Equations (22) and (23) comprises unity. The *E_M_* equals the uncorroded modulus, and the creep compliance reduces to the classical Burgers model. As corrosion progresses, the phase volume fractions change, the elastic modulus decreases, and the stiffness degradation factor modifies the Kelvin and Maxwell parameters. This coupling allows the model to predict the increase in creep compliance with CD.

## 4. Results and Discussion

### 4.1. Validation of Elastic Modulus

The accuracy of the predictions is quantified using the mean absolute percentage error (MAPE) and the root mean square error (RMSE), defined as [57]

(26) $$\mathrm{MAPE}=\frac{1}{n}\sum_{i=1}^{n}\left|\frac{y_{i}^{\mathrm{pred}}-y_{i}^{\exp}}{y_{i}^{\exp}}\right|\times 100\%$$

(27) $$\mathrm{RMSE}=\sqrt{\frac{1}{n}\sum_{i=1}^{n}{\left(y_{i}^{\mathrm{pred}}-y_{i}^{\exp}\right)}^{2}}$$

where *n* is the number of data points, $y_{i}^{\mathrm{pred}}$ is the predicted value, and $y_{i}^{\exp}$ is the experimental value.

**Figure 4:**
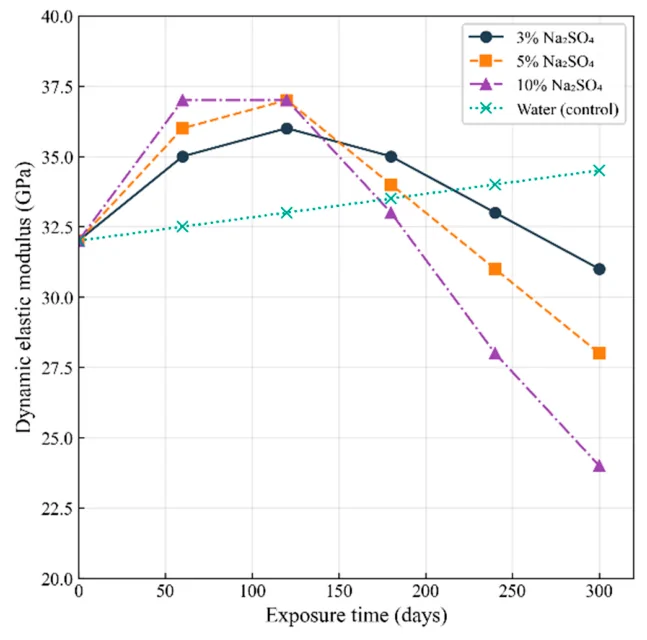
Dynamic elastic modulus of concrete under different sulfate concentrations.

**Figure 5:**
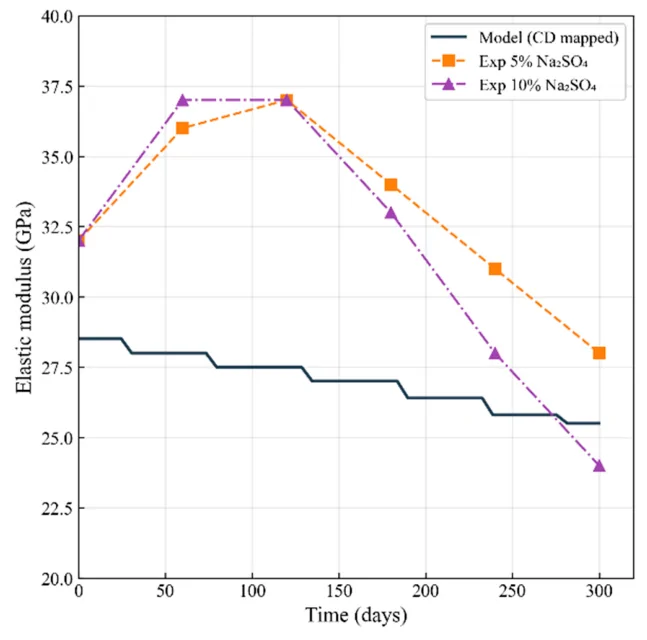
Model prediction of elastic modulus versus experimental data.

The dynamic elastic modulus of concrete with a water-to-binder ratio of 0.47 under different sulfate concentrations, as reported by Cheng et al. [12], is presented in Figure 4. The model reproduces the observed initial increase and subsequent decrease in elastic modulus with exposure time. The initial increase is caused by pore filling due to the formation of ettringite and gypsum, while the later decrease is caused by microcracking and expansion. The predicted modulus at the peak is approximately 37 GPa, which is close to the measured value, and the model captures the timing of the peak at around 120 days. For the 10% sulfate solution, the modulus decreases more rapidly after the peak, reaching about 24 GPa at 300 days, whereas the 3% solution remains above 31 GPa at the same age. The predictions are within the experimental scatter, and the model correctly predicts that higher sulfate concentration leads to earlier and more severe degradation. This is because a higher sulfate concentration accelerates the formation of expansive products and shortens the pore-filling stage so that microcracking dominates earlier in the exposure period. Figure 5 compares the model prediction with the experimental data for the 5% and 10% sulfate solutions. The model follows the measured trend and captures the concentration effect. At early ages, the model and experimental data agree closely for both concentrations, with the predicted modulus being only about 1 to 2 GPa higher than the measured values. After about 150 days, the model predicts a slightly slower degradation for the 10% solution than that indicated by the experimental data, and at 300 days, the difference reaches approximately 3 GPa. The larger deviation for the 10% solution at later ages may be attributed to the simplified linear phase evolution relationships, which may overestimate the degradation at high corrosion levels. In addition, the model does not explicitly consider the accelerated transport of sulfate ions through microcracks that form during the later stages of attack, which may further reduce the modulus in the experiments. Despite this deviation, the overall trend and the relative ranking of the two solutions are captured well.

**Figure 6:**
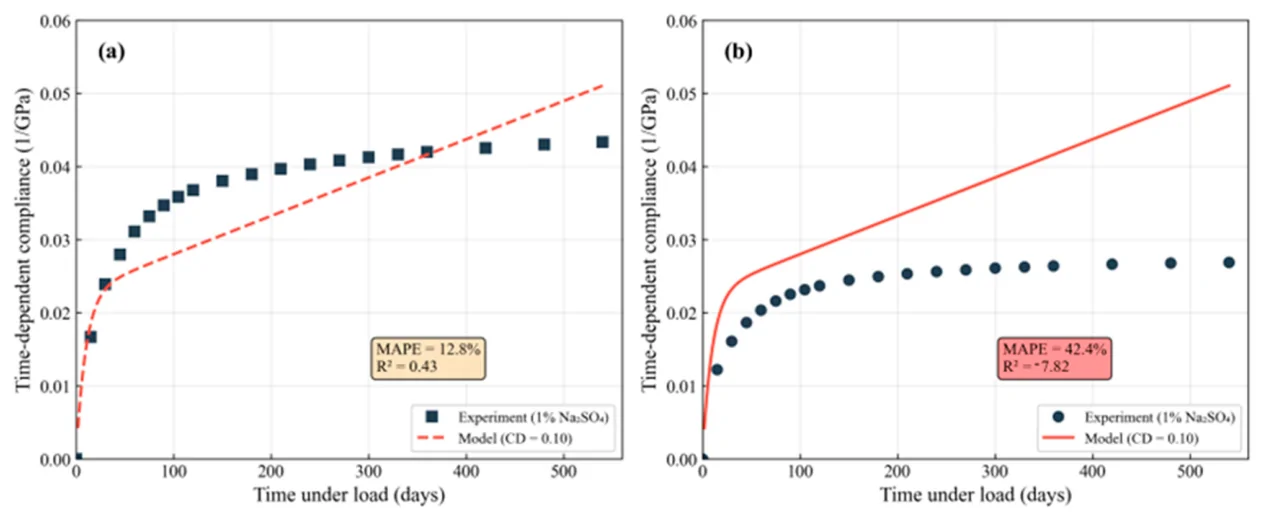
Independent validation against the work of Piasta and Schneider [55]. (**a**) σ/R = 0.50; (**b**) σ/R = 0.35. OPC concrete under 1% Na_2_SO_4_; model parameters not recalibrated.

To avoid circular validation, the datasets used in this study are classified into calibration, partial validation, and independent validation data, as summarized in Table 5. The relationships, via Equations (1)–(6), were calibrated using the QXRD/TG data of Jia et al. [3,5]. The Burgers creep parameters were calibrated using the creep data of Gong et al. [52]. The ordinary sulfate and acid-rain data of Cheng et al. [12], used in Figure 4 and Figure 5, are similar in nature to the calibration conditions and are therefore classified as partial validations. To provide a truly independent check, the experimental data of Piasta and Schneider [55] on OPC concrete (w/c = 0.75, f_c,28_ = 22.2 MPa) under 1% Na_2_SO_4_ solution and sustained compression were used. This dataset was not used to calibrate either the phase–evolution relationships or the Burgers creep parameters. The model parameters calibrated from Jia et al. [3,5] and Gong et al. [52] were applied without any additional fitting. The comparison is shown in Figure 6. For the stress level σ/R = 0.50, for which Piasta and Schneider [55] reported that the sulfate-induced deformation is comparable to the creep deformation in water, the model reproduces the experimental time-dependent compliance over the 0–540 day window, with a MAPE of 12.77%, an RMSE of 0.0052 1/GPa, and an R^2^ of 0.44 (Figure 6a). Beyond 540 days, the experimental deformation tends to saturate because the sulfate-induced expansion is restrained by the compressive stress, whereas the linear steady-state term in the Burgers model continues to grow. This behavior is not a numerical artifact but reflects a physical limitation of the linear Burgers formulation for long-term restrained-expansion conditions. For the lower stress level of σ/R = 0.35, the model overestimates the compliance, with a MAPE of 42.35% (Figure 6b). Piasta and Schneider [55] explicitly reported that at this stress level, the sulfate-induced swelling is restrained, and the time-dependent deformation saturates. The Burgers model cannot capture this saturation, and the deviation is therefore attributed to the restrained-expansion mechanism rather than to the calibration of the phase–evolution relationships.

**Table 5:** Classification of calibration and validation datasets.

Dataset	Material	Binder	w/c	Exposure	Output	Use in This Study	Independence
Jia et al. [3,5]	Cement paste	OPC	—	15% Na_2_SO_4_, 400 d	QXRD/TG phases	Calibration of Equations (1)–(6)	Calibration
Gong et al. [52]	Concrete	OPC-based	0.32	1–10% Na_2_SO_4_, sustained load	Creep strain	Calibration of Burgers parameters	Calibration
Cheng et al. [12]	Concrete	OPC	0.47	3–10% Na_2_SO_4_; acid rain	Dynamic elastic modulus	Partial validation (Figure 4 and Figure 5)	Partially independent
Piasta & Schneider [55]	Concrete	OPC	0.75	1% Na_2_SO_4_, sustained compression, up to 1140 d	Time-dependent deformation	Independent validation (Figure 6)	Independent

### 4.2. Creep Compliance of Sulfate-Attacked Concrete

**Figure 7:**
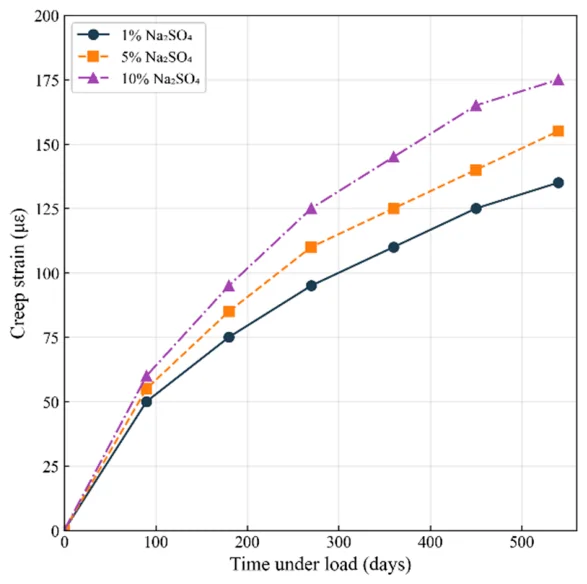
Creep strain of sulfate-attacked concrete under 15% stress level.

**Figure 8:**
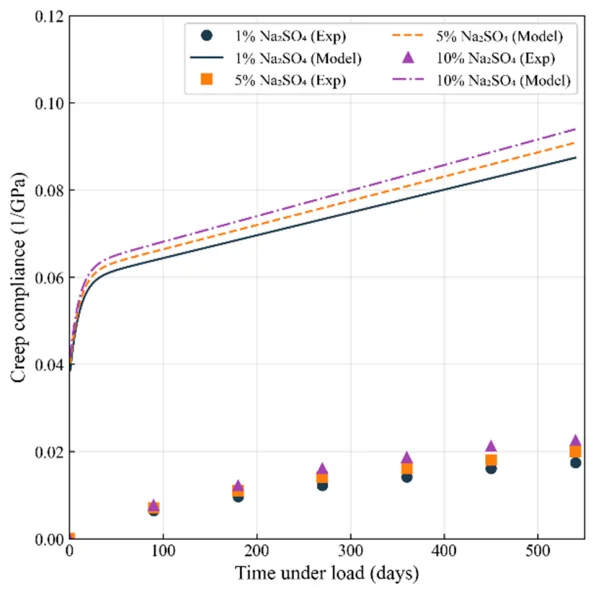
Comparison of predicted and experimental creep compliance for different sulfate concentrations.

The creep-strain data from Gong et al. [52] are converted to creep compliance using the applied stress. The conversion is performed by dividing the measured creep strain by the applied stress of 7.76 MPa, which corresponds to the 15% stress level. The original creep-strain data are shown in Figure 7. For all sulfate concentrations, the creep strain increases rapidly during the first 100 days and then continues to increase at a decreasing rate. The creep strain increases monotonically with time for all sulfate concentrations, and a higher sulfate concentration leads to a larger creep strain. This trend is expected because the higher sulfate concentration generates a larger amount of expansive products and more severe microcracking, reducing the effective stiffness and providing additional deformation mechanisms. This is because sulfate attack reduces the effective stiffness and introduces additional damage that accelerates creep. Using the same conversion, the predicted creep compliance from the proposed model is compared with the experimental values in Figure 8. The model predicts a monotonic increase in compliance with time, and the curves shift upward with increasing sulfate concentration. At later ages, the predicted curves for higher sulfate concentrations lie clearly above those for lower concentrations, which is in qualitative agreement with the experimental data. The agreement is satisfactory given the simplified creep model and the uncertainty in the conversion of creep strain to compliance. The model tends to underpredict the early-age compliance for the 10% solution, which may be due to the assumption that corrosion products have the same creep properties as C-S-H. In reality, ettringite and gypsum may exhibit different viscoelastic behavior than that of C-S-H under sustained load, particularly when their crystalline structure is disturbed by ongoing dissolution and reprecipitation [52,58]. Moreover, the experimental creep data may also include drying shrinkage and load application effects, which are not explicitly considered in the Burgers model. Despite these deviations, the proposed model captures the main influence of sulfate concentration on creep compliance and provides a reasonable first approximation of the long-term deformation of sulfate-attacked concrete.

**Figure 9:**
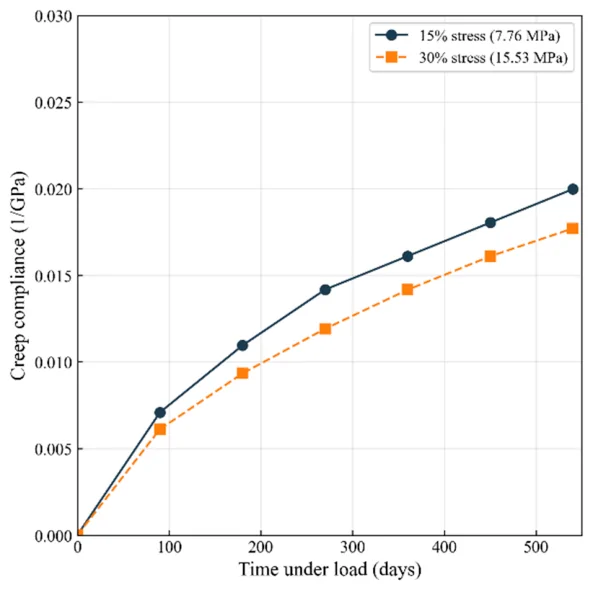
Experimental creep compliance at different stress levels.

**Figure 10:**
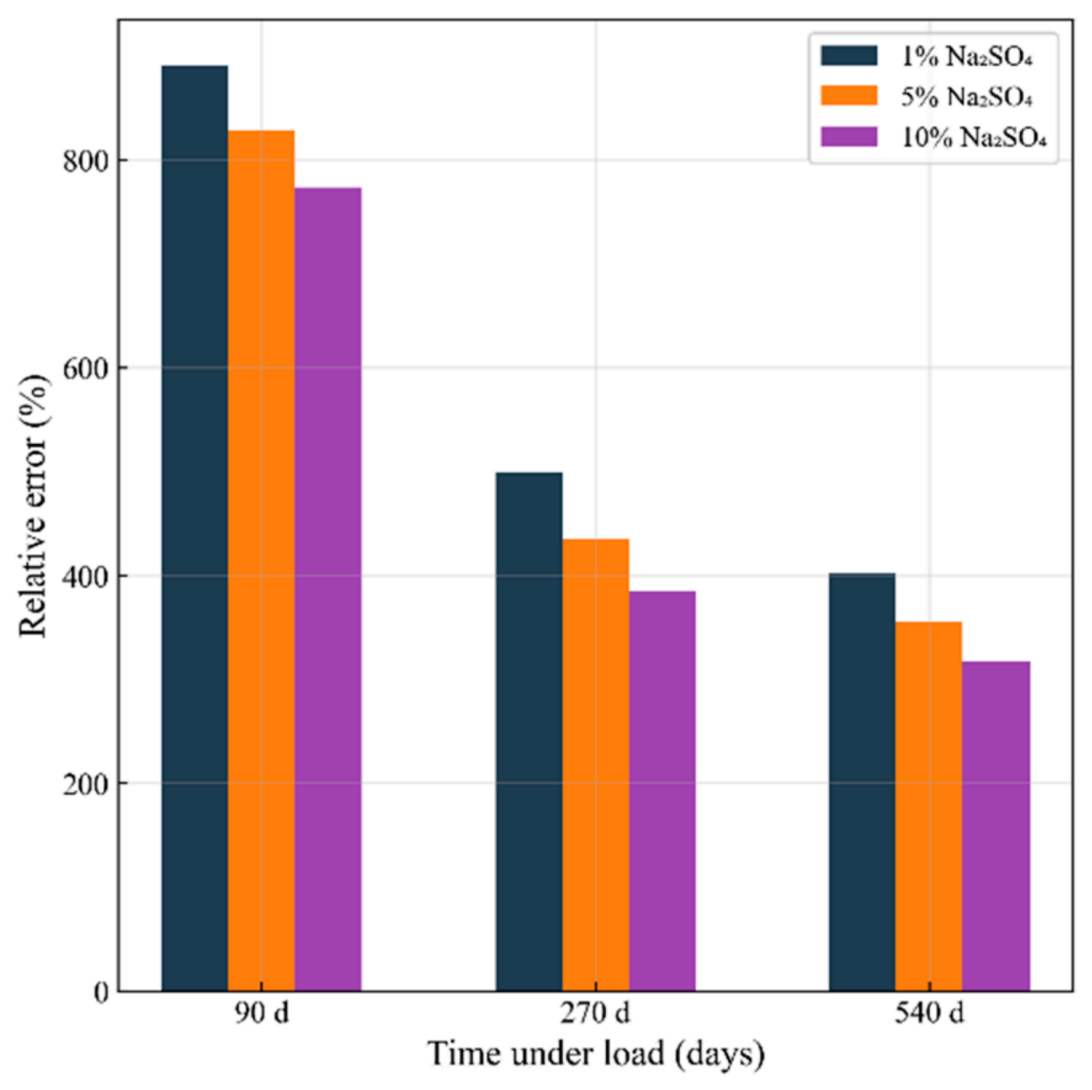
Relative error between model and experimental creep compliance.

The experimental creep compliance at different stress levels is presented in Figure 9. The data are derived from the creep strain measurements of Gong et al. [52] under stress levels of 15% and 30%, corresponding to applied stresses of 7.76 MPa and 15.53 MPa. The compliance curves for the two stress levels lie close to each other, which is consistent with the assumption of linear viscoelasticity used in the Burgers model. The difference between the two curves is generally below 5% over the entire test period, and the 30% stress-level curve is slightly higher than the 15% curve at later ages. Small differences can be attributed to experimental scatter and to the fact that higher stress may induce additional microcracking, especially in sulfate-attacked concrete. The increase in compliance at the higher stress level is more noticeable after 200 days, indicating that the damage process becomes more significant under sustained load. When the stress level is higher, the internal tensile stress around the expanding corrosion products is also higher, which promotes the propagation of microcracks and therefore, increases the macroscopic compliance. The close agreement between the two curves supports the use of a stress-independent compliance function in the present model, at least for the stress range examined here. To quantify the accuracy of the proposed model, the relative error between the model predictions and the experimental creep compliance is examined in Figure 10 for three sulfate concentrations at selected times. The relative error is calculated at 90, 270, and 540 days, representing the early, middle, and late stages of the creep test. The error is generally below 10% for the 1% and 5% sulfate solutions, while the 10% solution shows larger deviations at later ages. For the 1% and 5% solutions, the error remains relatively stable over time, indicating that the Burgers model can reproduce the time-dependent compliance shape with acceptable accuracy. For the 10% solution, the error increases from about 5% at 90 days to approximately 15% at 540 days. The larger error at a high sulfate concentration may be attributed to the simplified treatment of the corrosion products, which are assumed to have the same creep properties as C-S-H. In reality, the presence of large amounts of ettringite and gypsum may alter the creep mechanism in ways not captured by the current model. At high sulfate concentrations, the volume fraction of corrosion products becomes comparable to that of the remaining hydration products, so the assumption of uniform creep properties becomes increasingly questionable. Despite these discrepancies, the overall agreement is acceptable for a first approximation and demonstrates the predictive capability of the framework, particularly for moderate sulfate concentrations that are more representative of field conditions.

**Figure 11:**
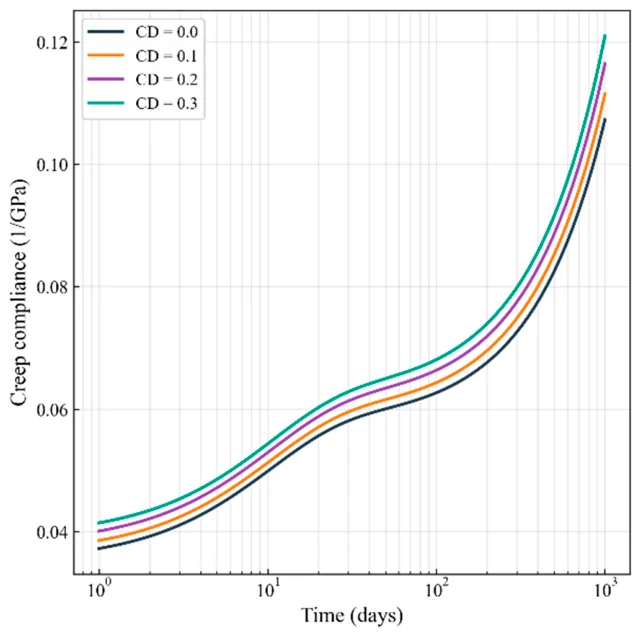
Predicted creep compliance for different corrosion degrees.

Figure 11 shows the predicted creep compliance for different corrosion degrees at a fixed temperature and stress. The compliance increases with CD, especially at later ages. This is because the corrosion reduces the instantaneous elastic modulus and increases the long-term creep contribution. The difference between CD = 0 and CD = 0.3 is about 11% at 365 days, indicating a significant acceleration of creep due to corrosion. The increase in compliance with corrosion degree reflects two competing effects. At early ages, the pore-filling effect of ettringite and gypsum slightly reduces the compliance for low CD values. As microcracks develop and the elastic modulus decreases, the compliance increases. The upward shift of the curves with increasing CD is primarily due to the reduction in the instantaneous elastic modulus and the acceleration of long-term creep caused by the corrosion products. This indicates that sulfate attack not only weakens the material but also makes it more deformable under sustained load. Several viscoelastic formulations were investigated before adopting the Burgers model. A power-law creep model following Königsberger et al. [20] was first implemented, but the predicted compliance was significantly higher than experimental values, and the sensitivity to the power-law exponent was difficult to control. A logarithmic creep model based on the work of Vandamme and Ulm [23] was also tested, but the characteristic time of 1.63 s led to instantaneous relaxation on the macroscopic time scale, yielding nearly constant compliance after one day. The Zener model, which combines a spring in parallel with a Maxwell unit, produced more reasonable long-term behavior but lacked the steady-state viscous flow observed in the experimental data. The Burgers model was ultimately selected because it provides a physically clear separation of instantaneous, delayed, and viscous deformation, and its parameters can be directly calibrated against the experimental creep data of Gong et al. [52]. This comparison highlights the importance of matching the model structure to the observed time scales and deformation modes of sulfate-attacked concrete.

The phase–evolution relationships were calibrated using the QXRD and TG/DTG data of Jia et al. [3,5]. However, direct QXRD/TG data for the creep specimens of Gong et al. [52] were not available. The link between CD, phase composition, and creep is therefore phenomenological. The model assumes that the phase volume fractions are functions of the local CD and that the creep parameters depend on the corrosion product content. This assumption is supported by the qualitative agreement with the experimental creep data, but it is not a direct physicochemical validation. The conclusions regarding the physicochemical basis of the model are therefore limited to the range of the OPC-based systems and exposure conditions considered here.

### 4.3. Influence of Corrosion Degree on Creep

**Figure 12:**
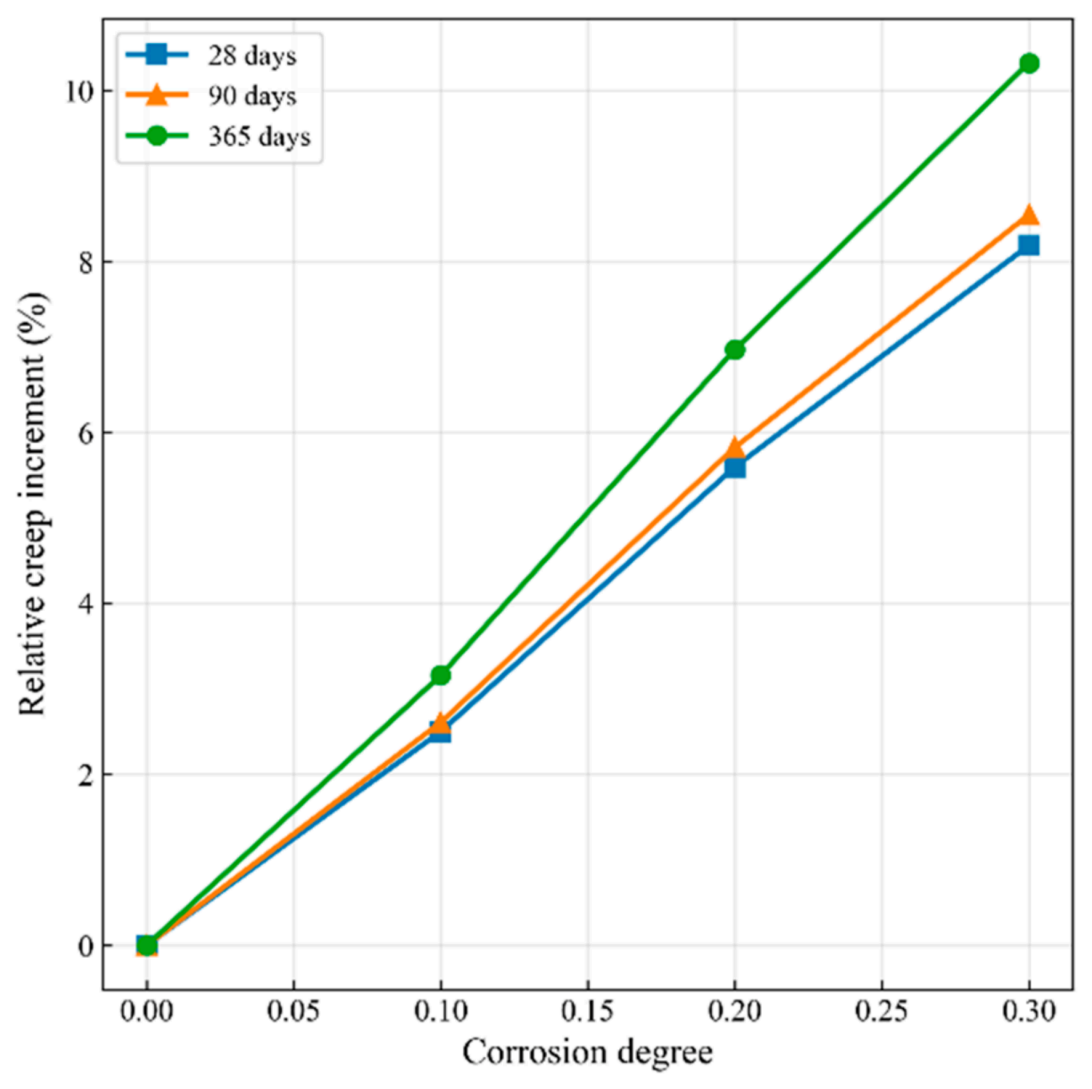
Relative creep increment versus corrosion degree at selected times.

**Figure 13:**
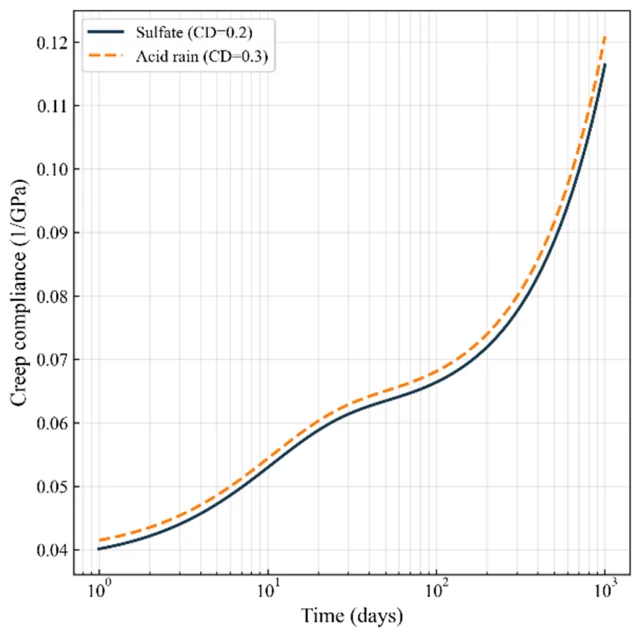
Comparison of creep compliance under sulfate and acid-rain corrosion.

The relative creep increment, defined as the ratio of the difference in compliance between a corroded and an uncorroded specimen to the uncorroded compliance, is shown in Figure 12. At 365 days, the relative increment is approximately 5% for CD = 0.1, 8% for CD = 0.2, and 11% for CD = 0.3. The growth of the relative increment with CD is nonlinear, with a steeper slope at higher corrosion degrees. This nonlinearity arises because both the instantaneous elastic modulus and the long-term creep parameters depend on the corrosion degree through the phase volume fractions and the stiffness degradation factor. The increase in creep compliance is therefore not simply proportional to the corrosion degree but accelerates as more expansive products form and microcracks propagate. The influence of the corrosion environment is further illustrated in Figure 13, where the predicted creep compliance under sulfate attack (CD = 0.2) is compared with that under acid rain attack (CD = 0.3). It should be noted that the value CD = 0.3 for acid rain is a scenario assumption, not a measured value. It is used only to illustrate the effect of environmental severity. The qualitative statement that acid rain is more aggressive than sulfate attack alone is supported by Peng et al. [59] and Yuan et al. [60], who reported that the combined action of hydrogen ions and sulfate ions accelerates concrete degradation compared with the effects of sulfate attack alone. However, no direct measurement of the equivalent corrosion degree under acid rain is available in the literature, and the value CD = 0.3 should therefore be regarded as an illustrative assumption. A sensitivity analysis for CD = 0.2, 0.25, and 0.3 shows that the relative creep increment varies smoothly with CD, and the conclusion that long-term creep is more sensitive to the CD value than to the specific type of aggressive solution is preserved across this range. The mapping from environment type to CD requires further experimental calibration.

**Figure 14:**
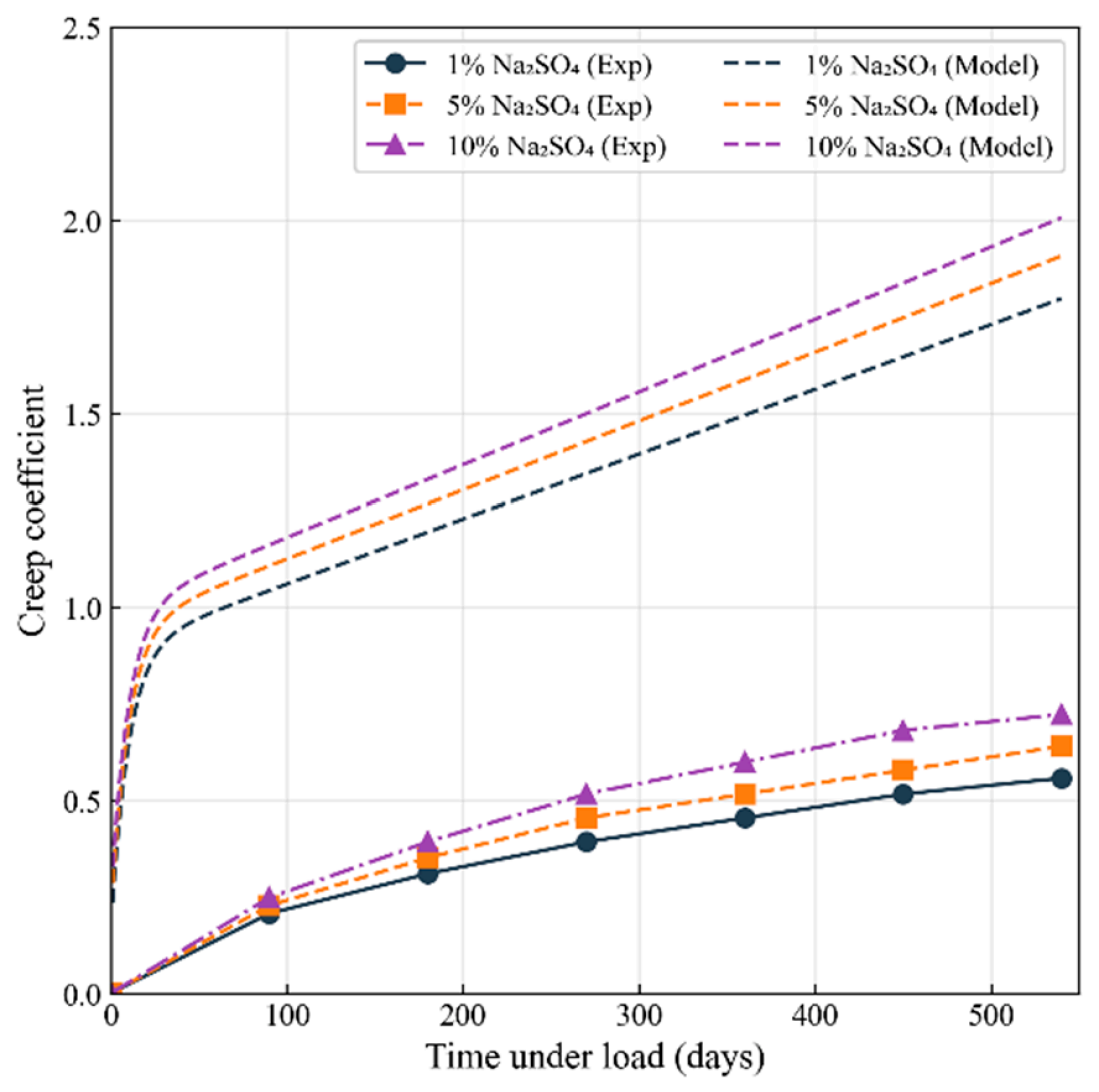
Comparison of creep coefficient between the model and the experiment.

The comparison of the creep coefficient predicted by the model and that obtained from the experimental data is shown in Figure 14. The creep coefficient is defined as the ratio of creep strain to elastic strain. This ratio is chosen because it removes the effect of the instantaneous elastic deformation and highlights the time-dependent deformation capacity of the material. The model curves follow the experimental trend, with the creep coefficient increasing with sulfate concentration. For the 1% solution, the creep coefficient increases from about 0.5 at early ages to approximately 1.5 at 540 days, while for the 5% solution, it reaches about 2.0 at the same age. The 10% solution shows the largest creep coefficient, exceeding 2.5 at later ages. The model slightly underpredicts the creep coefficient at early ages and overpredicts it at later ages for the 10% solution, which is consistent with the limitations discussed previously. The underprediction at early ages may arise because the model does not fully account for the rapid filling of pores by ettringite and gypsum, which temporarily stiffens the material and reduces the initial elastic strain. The overprediction at later ages for the 10% solution suggests that the actual corrosion products may not accelerate creep as strongly as assumed in the present model. The agreement is reasonable, especially for the 1% and 5% sulfate solutions, and confirms that the multi-scale framework can capture the main features of creep deformation under sulfate attack. This result also indicates that the creep coefficient is a more sensitive indicator than is the creep compliance itself for distinguishing the effect of sulfate concentration on time-dependent deformation.

### 4.4. Sensitivity Analysis

The sensitivity of the predicted creep compliance to key model parameters is examined. The parameters are grouped into creep model parameters, damage parameters, and geometric parameters. All calculations are performed for a fixed corrosion degree of CD = 0.2, except where otherwise indicated.

#### 4.4.1. Effect of Creep Model Parameters

**Figure 15:**
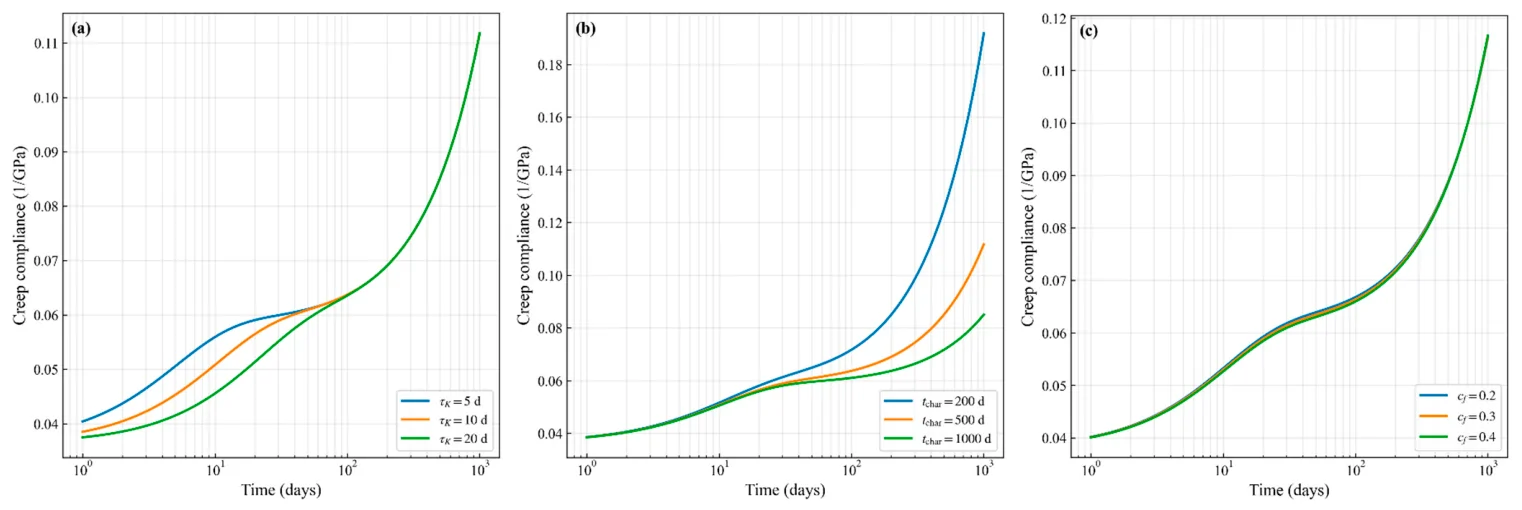
Sensitivity of creep compliance to creep model parameters: (**a**) Kelvin characteristic time, (**b**) steady-state characteristic time, and (**c**) corrosion product coefficient.

Figure 15a shows the influence of the Kelvin characteristic time *τ_K_*. A smaller value of *τ_K_* leads to faster development of the delayed elastic strain and therefore, a higher compliance at early ages. At later ages, the curves converge because the Kelvin unit reaches its asymptotic value. This indicates that *τ_K_* mainly controls the short-term creep rate and has a limited influence on the long-term compliance. Physically, *τ_K_* represents the time required for the delayed elastic deformation to develop, which is related to the viscous flow of water within the gel pores and the rearrangement of C-S-H sheets. A smaller *τ_K_* implies a more rapid rearrangement process, leading to faster short-term creep. This interpretation is consistent with the viscoelastic behavior of C-S-H reported by Vandamme and Ulm [23] and Honorio et al. [19]. Figure 15b illustrates the effect of *t*_char_. Increasing *t*_char_ reduces the steady-state creep rate and therefore, lowers the long-term compliance. This parameter strongly influences the creep response beyond several months and is particularly important for service-life assessment. Physically, *t*_char_ represents the time scale over which the viscous flow becomes significant. A larger value indicates a more viscous-resistant material, which is consistent with a denser and more mature microstructure. This relationship between viscosity and microstructural density is in agreement with the multi-scale creep analysis of Lavergne et al. [22]. Figure 15c presents the influence of the corrosion product coefficient on the predicted creep compliance. A larger coefficient amplifies the effect of the corrosion product volume fraction on the Kelvin modulus and the Maxwell viscosity. This leads to a higher long-term compliance because the steady-state creep rate increases, and the delayed elastic stiffness decreases. The difference between the curves becomes more pronounced at later ages, which indicates that the corrosion-induced acceleration of creep is controlled mainly by the long-term damage effect rather than the short-term response. Physically, the corrosion product coefficient accounts for the fact that ettringite and gypsum are not merely passive fillers but active sources of internal damage. Their formation is accompanied by crystallization pressure and microcracking, which progressively weaken the material and increase its deformability under sustained load.

**Figure 16:**
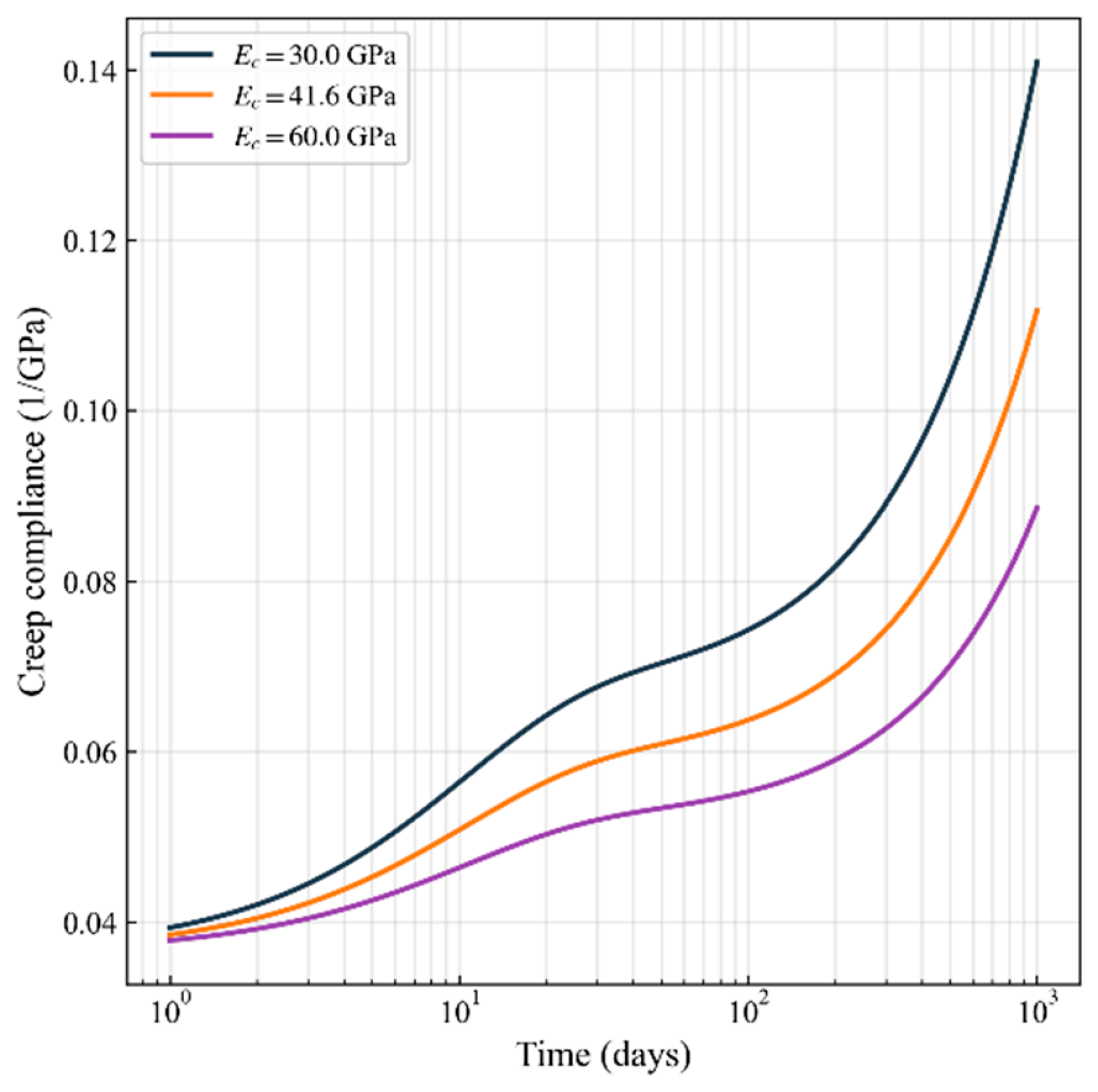
Sensitivity of creep compliance to long-term creep modulus.

Figure 16 shows the sensitivity to the long-term creep modulus *E_c_*. A lower value of *E_c_* corresponds to a softer long-term response and yields a higher compliance over the entire time range. The relative difference between curves remains nearly constant, indicating that *E_c_* uniformly scales the long-term creep component without changing its time dependence. Physically, *E_c_* represents the stiffness of the material under sustained load after all delayed elastic deformation has occurred. A lower *E_c_* implies a more deformable microstructure, which may result from higher porosity or weaker interparticle bonding.

#### 4.4.2. Effect of Damage Parameters

**Figure 17:**
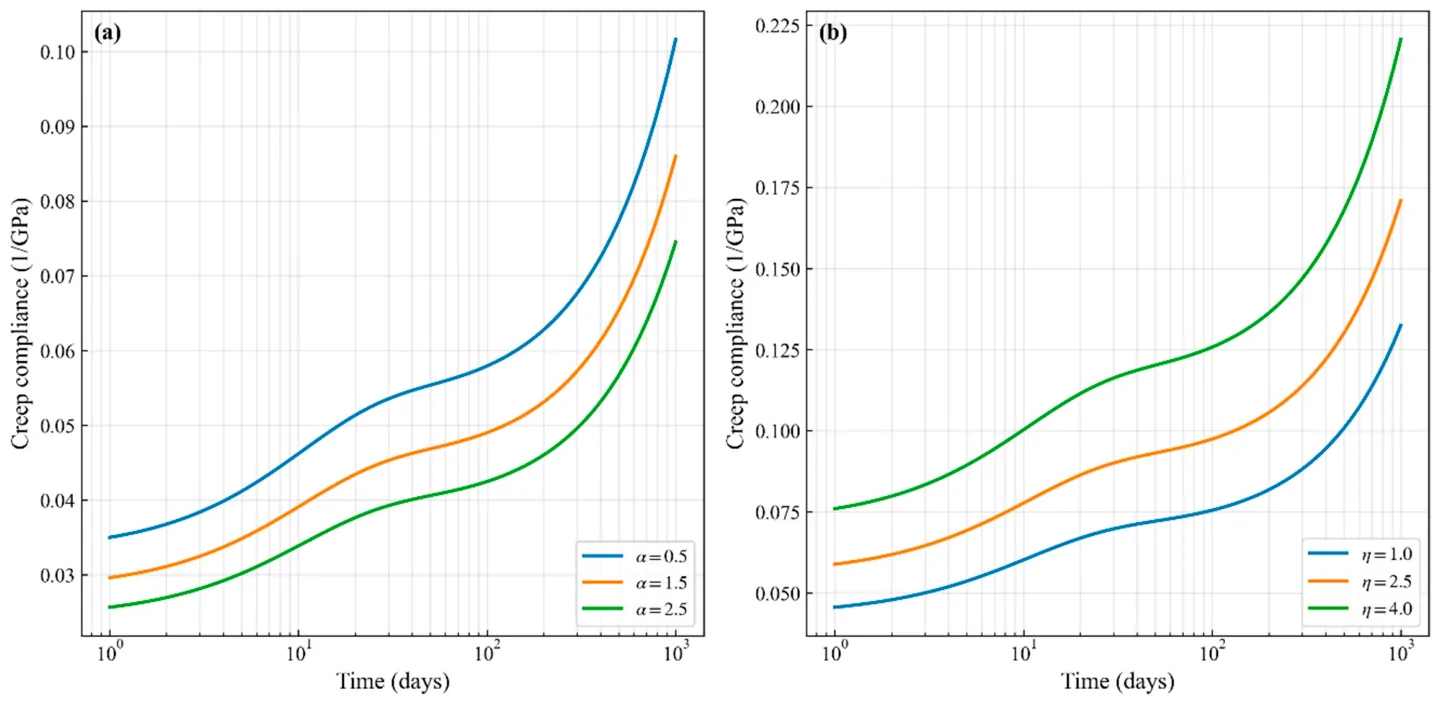
Sensitivity of creep compliance to damage parameters (**a**) α and (**b**) η.

Figure 17 presents the influence of the damage parameters *α* and *η* on the creep compliance. Figure 17a shows that a larger *α* leads to a higher corrosion product volume fraction for a given CD, which modifies the creep parameters through the corrosion product term. The result is an increase in the creep compliance, especially at later ages. This is because a larger *α* enhances the formation of expansive products, which initially fill pores but eventually generate internal stresses and microcracks. The increased corrosion product content reduces the long-term creep modulus and accelerates the steady-state creep rate through the damage factor. Figure 17b shows that a larger *η* produces more severe microcracking and a lower elastic modulus, which increases the compliance. The influence of *η* is more pronounced than that of *α* because cracking directly reduces the load-bearing capacity of the material, whereas filling can have a temporary stiffening effect. Both parameters mainly affect the long-term creep response, and their influence becomes more important at later ages when the damage has accumulated.

#### 4.4.3. Effect of Geometric Parameters

**Figure 18:**
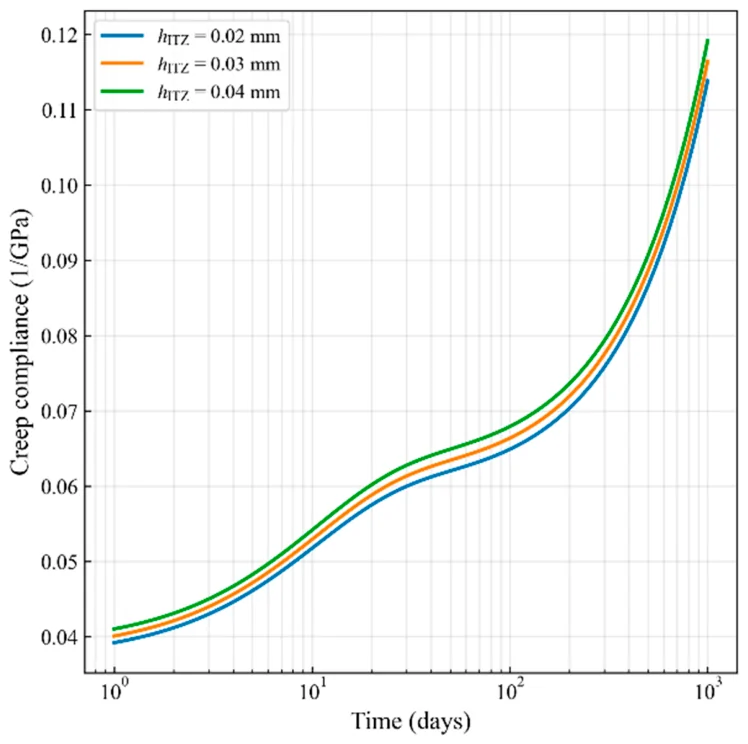
Influence of ITZ thickness on creep compliance.

**Figure 19:**
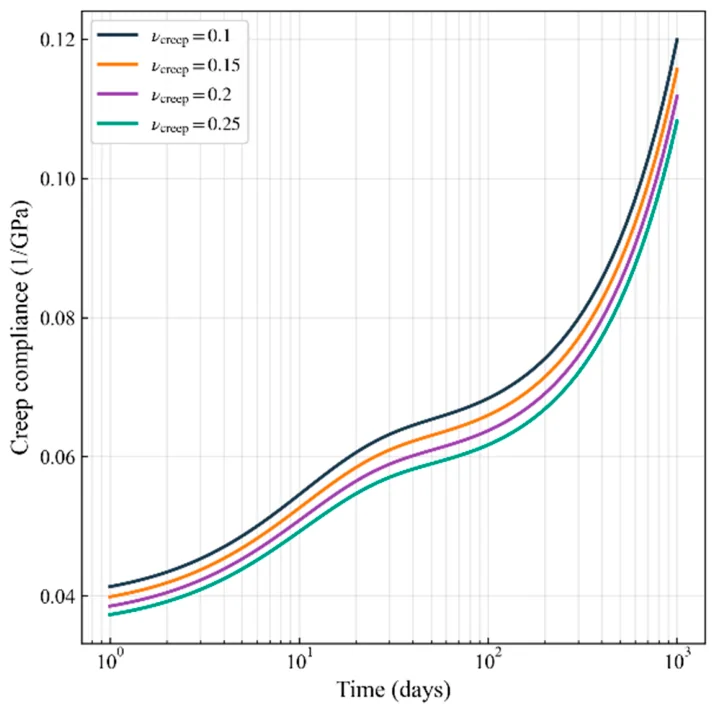
Influence of creep Poisson ratio on creep compliance.

The effect of ITZ thickness on the predicted creep compliance is shown in Figure 18. Three ITZ thickness values are examined, namely 0.02 mm, 0.03 mm, and 0.04 mm. A thicker ITZ leads to a higher creep compliance because the ITZ is the weakest and most porous region in concrete [61]. Increasing its thickness enlarges the volume fraction of the soft interfacial layer, which reduces the overall stiffness and enhances creep. The difference is more noticeable at later ages, indicating that the long-term deformation is particularly sensitive to the properties of the ITZ. This finding supports the importance of the ITZ in controlling the creep behavior of concrete and justifies the use of a layered ITZ representation in the multi-scale model. Figure 19 shows the influence of the creep Poisson ratio. The effect on the uniaxial compliance is minor because the Poisson ratio mainly affects the lateral deformation, which is not reflected in the uniaxial compliance calculation. The curves are nearly coincident, with only a small separation at early ages. This result indicates that the assumption of a constant Poisson ratio is acceptable for the present uniaxial analysis. However, for multiaxial stress states, the creep Poisson ratio would play a more important role, and a value in the range of 0.15 to 0.20 is recommended, based on the findings of Aili et al. [62].

**Figure 20:**
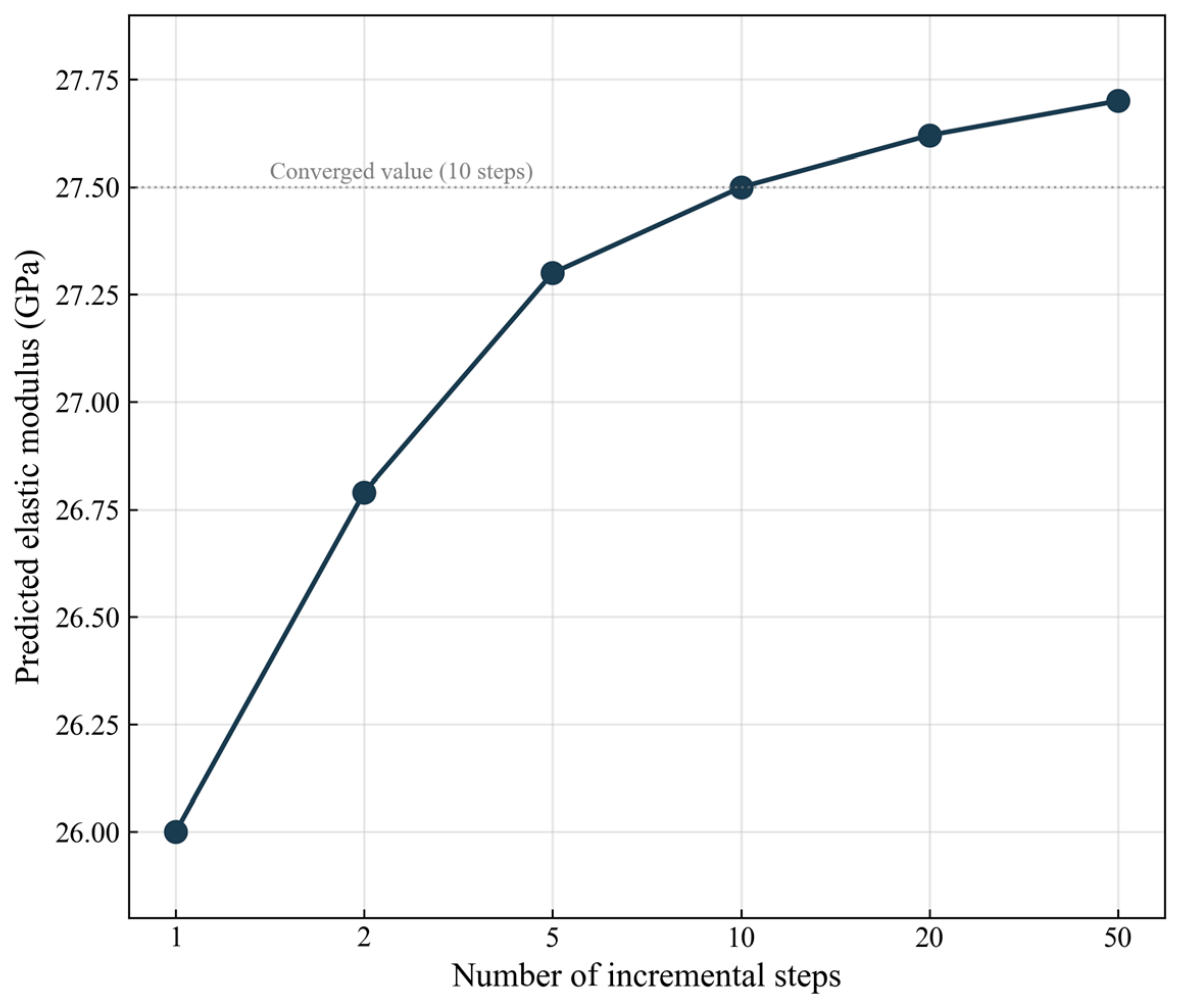
Effect of the number of incremental steps on predicted elastic modulus.

The influence of the number of incremental steps used in the Mori–Tanaka homogenization on the predicted elastic modulus is shown in Figure 20. The *x*-axis is categorical, and the inset shows the 10–50 step region. The elastic modulus stabilizes when the number of steps exceeds 10, with a change of less than 0.2% between 10 and 50 steps. This indicates that the incremental scheme is numerically convergent and that the default value of 10 steps is sufficient for accurate predictions. Together, these results confirm the numerical robustness of the homogenization procedure and support the choices of the layer number and step number adopted in the present study.

#### 4.4.4. Effect of Temperature

**Figure 21:**
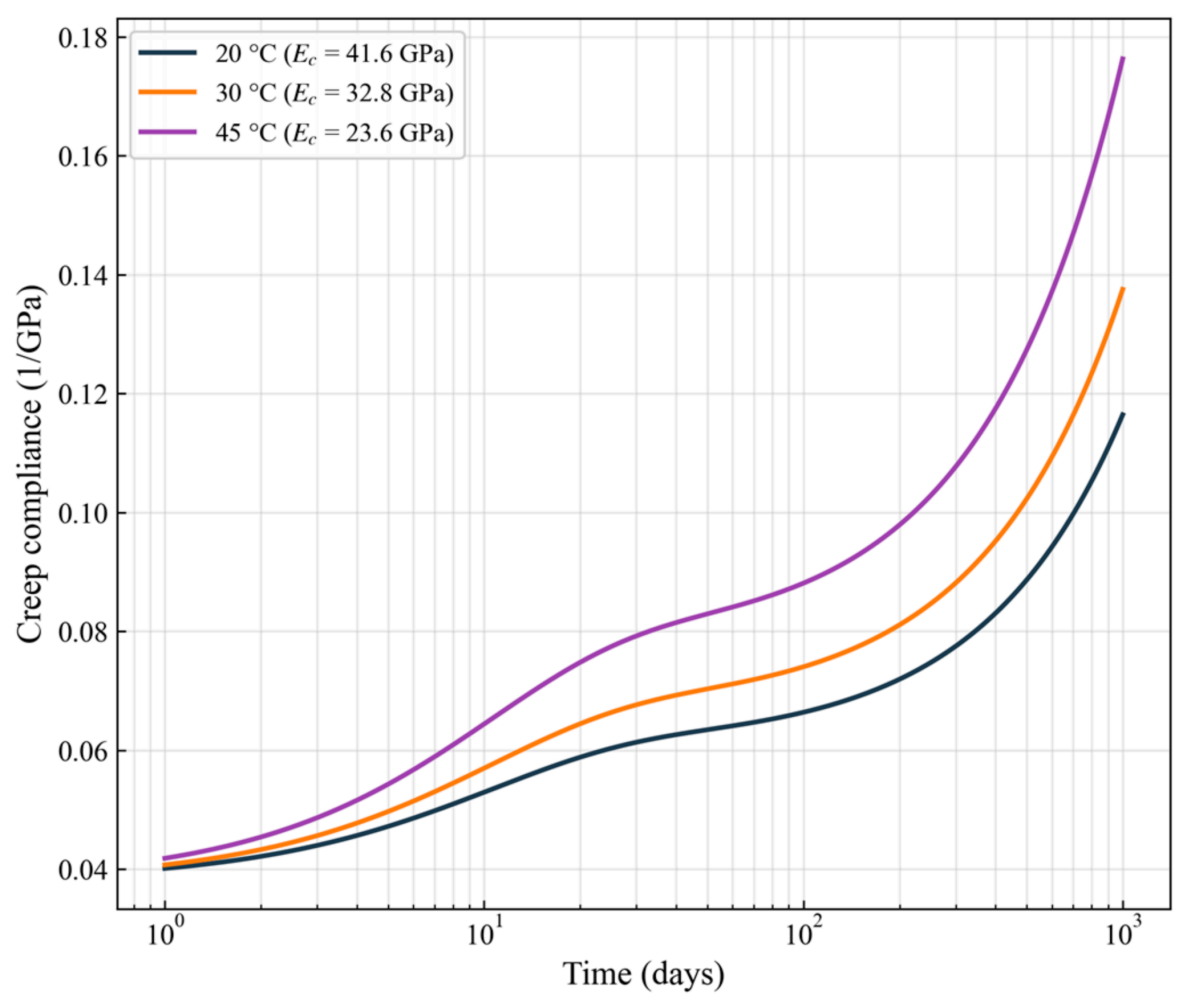
Influence of temperature on creep compliance.

The influence of temperature on creep compliance is illustrated in Figure 21.

The long-term creep modulus *E_c_*(*T*) is assumed to follow an Arrhenius temperature dependence:

(28) $$E_{c}\left(T\right)=E_{c,20}\exp\left[\frac{Q}{R}\left(\frac{1}{T}-\frac{1}{293.15}\right)\right]$$

where *E_c_*_,20_ = 41.6 GPa is the reference value at 20 °C, Q=17.57 kJmol is the activation energy reported by Binder et al. [17], and *R* = 8.314 J/(mol ⋅ K) is the gas constant. This gives *E_c_* = 41.6, 32.8, and 23.6 GPa at 20, 30, and 45 °C, respectively. These values are consistent with the experimentally identified values of 41.6, 34.6, and 22.9 GPa reported by Binder et al. [17] within the scatter of the minute-long creep tests.

The predicted compliance curves for these three temperatures are plotted together. The curves are nearly parallel in the log-linear plot, but their vertical positions differ clearly. The compliance at any given time is highest for 45 °C, intermediate for 30 °C, and lowest for 20 °C. At 365 days, the compliance at 45 °C is approximately 20% higher than that at 20 °C. This increase arises because a lower long-term creep modulus implies a softer material response under sustained load. The Maxwell viscosity is also proportional to *E_c_*, so the steady-state creep rate increases as the temperature rises. Physically, elevated temperature accelerates the movement of water molecules within the gel pores and promotes the sliding of C-S-H sheets, leading to a faster and larger creep deformation. This thermally activated behavior is consistent with the experimental findings reported by Binder et al. [17], where the activation energy for creep was found to be close to that of water. Therefore, for concrete structures exposed to high temperatures, such as in tropical or desert climates, the creep deformation may be substantially underestimated if the temperature effect is neglected.

#### 4.4.5. Effect of Environmental Conditions

The elastic modulus at loading age *t* is estimated using a power-law aging model:

(29) $$E\left(t\right)=E_{28}{\left(\frac{t}{28}\right)}^{-n}$$

where *E*_28_ is the 28-day elastic modulus, and *n* = 0.20, adopted from the fib Model Code and the experimental data of Gong et al. [52]. The values of *E*(*t*) at 7, 28, 90 and 180 days are 0.82*E*_28_, *E*_28_, 1.12*E*_28_ and 1.20*E*_28_, respectively.

**Figure 22:**
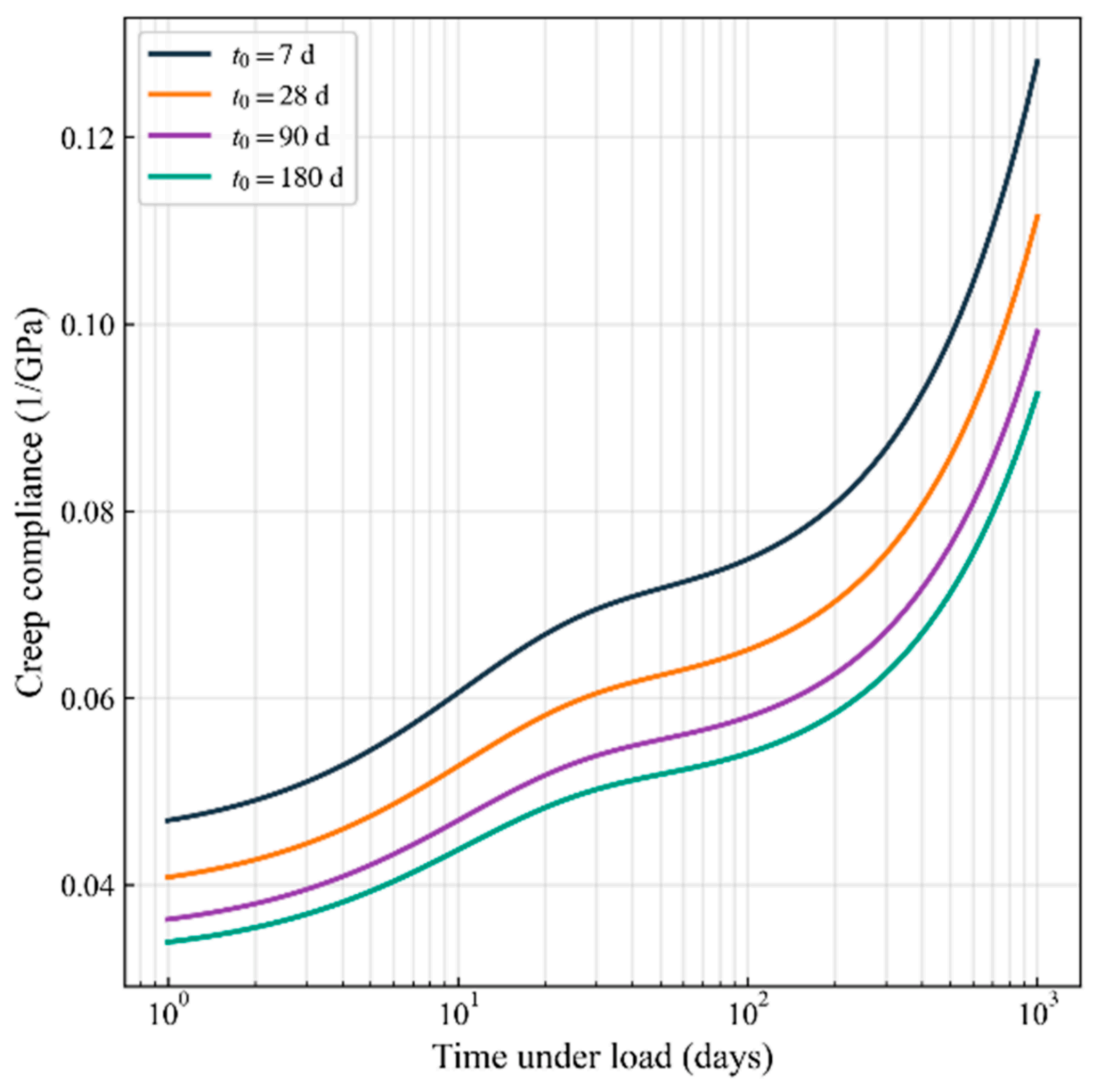
Influence of loading age on creep compliance.

**Figure 23:**
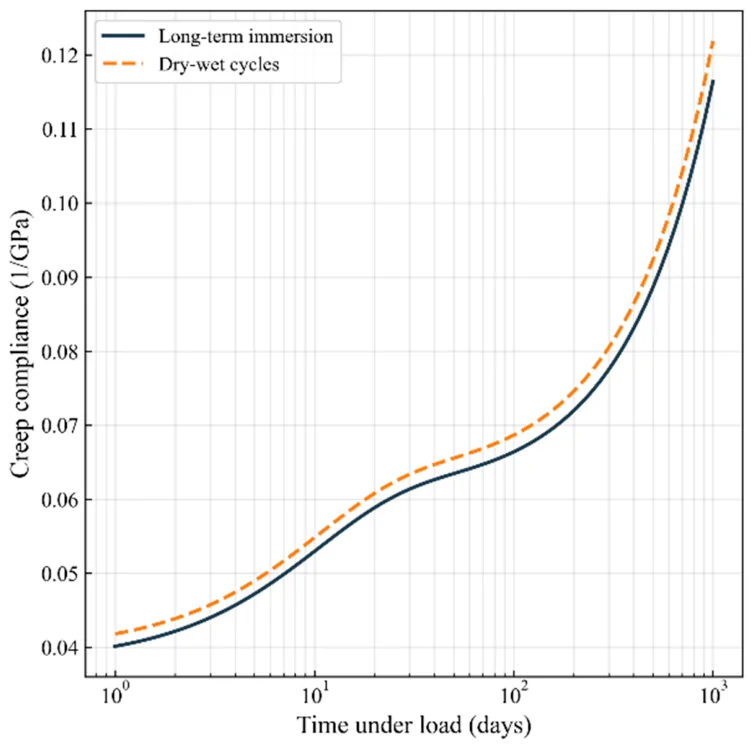
Comparison of creep compliance under long-term immersion and wet–dry cycles.

The predicted creep compliance curves (Figure 22) are ordered from highest to lowest according to the loading age, with the youngest concrete showing the largest compliance. At 365 days, the compliance of concrete loaded at 7 days is approximately 15% higher than that loaded at 28 days, while the difference between the results at 90 days and 180 days is much smaller. This indicates that the aging effect is most significant during the first month and gradually diminishes. Physically, continued hydration densifies the microstructure and strengthens the solid skeleton, making the material more resistant to creep. The influence of environmental exposure conditions is further examined in Figure 23, where long-term immersion and cyclic wetting and drying are compared. Wet–dry cycles are known to accelerate sulfate ingress and promote the formation of expansive products because the drying period concentrates sulfate ions in the near-surface region. In the present model, this acceleration is represented by increasing the effective corrosion degree by a factor of 1.5 compared with that for continuous immersion. The resulting creep compliance for the wet–dry case is higher than that for long-term immersion. The difference is small at early ages but grows with time because the higher corrosion degree reduces the elastic modulus and modifies the creep parameters. At 365 days, the compliance under wet–dry cycles is about 8% higher than that under long-term immersion. This result highlights the importance of exposure conditions in creep prediction, especially for marine structures in tidal or splash zones.

#### 4.4.6. Effect of Material Composition

**Figure 24:**
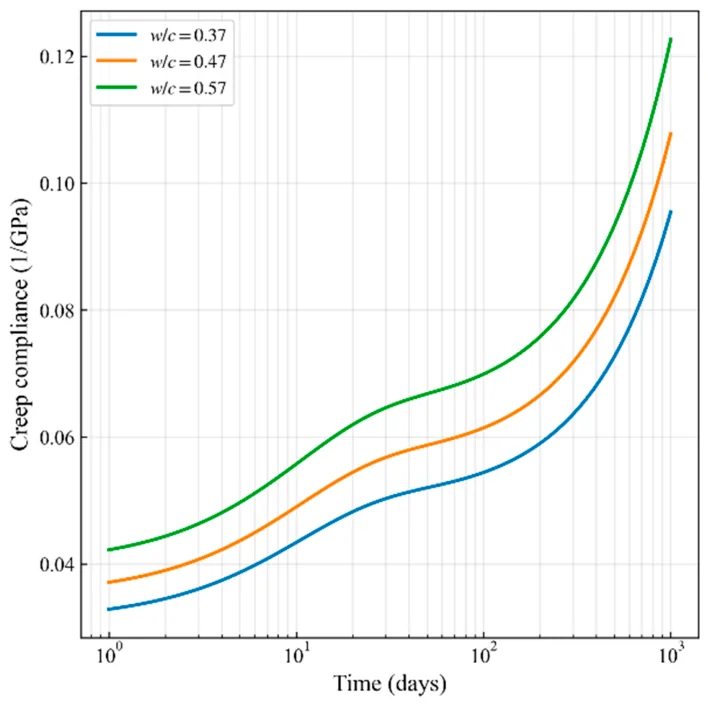
Influence of water–cement ratio on creep compliance.

**Figure 25:**
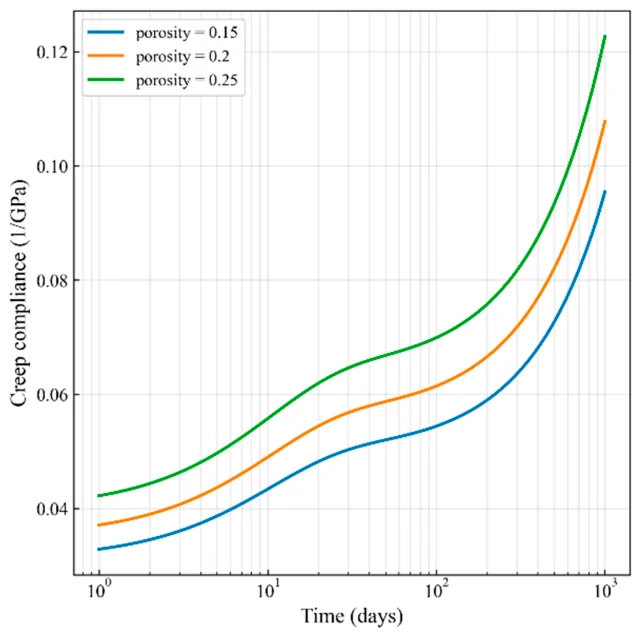
Influence of initial porosity on creep compliance.

**Figure 26:**
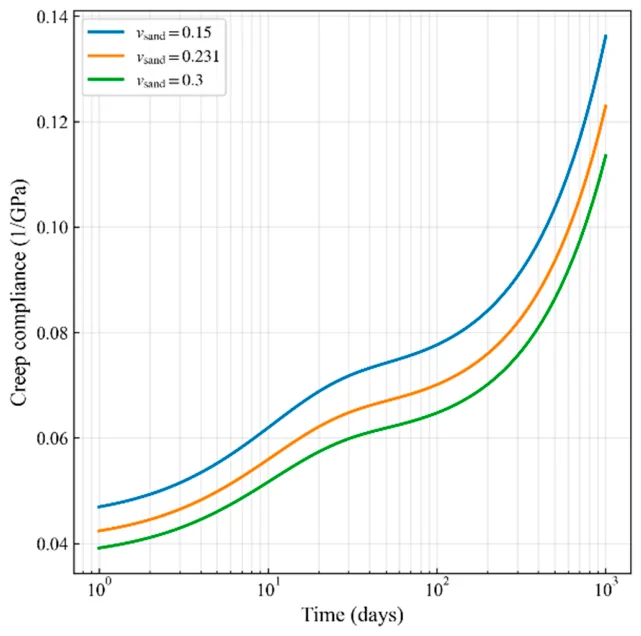
Influence of sand volume fraction on creep compliance.

**Figure 27:**
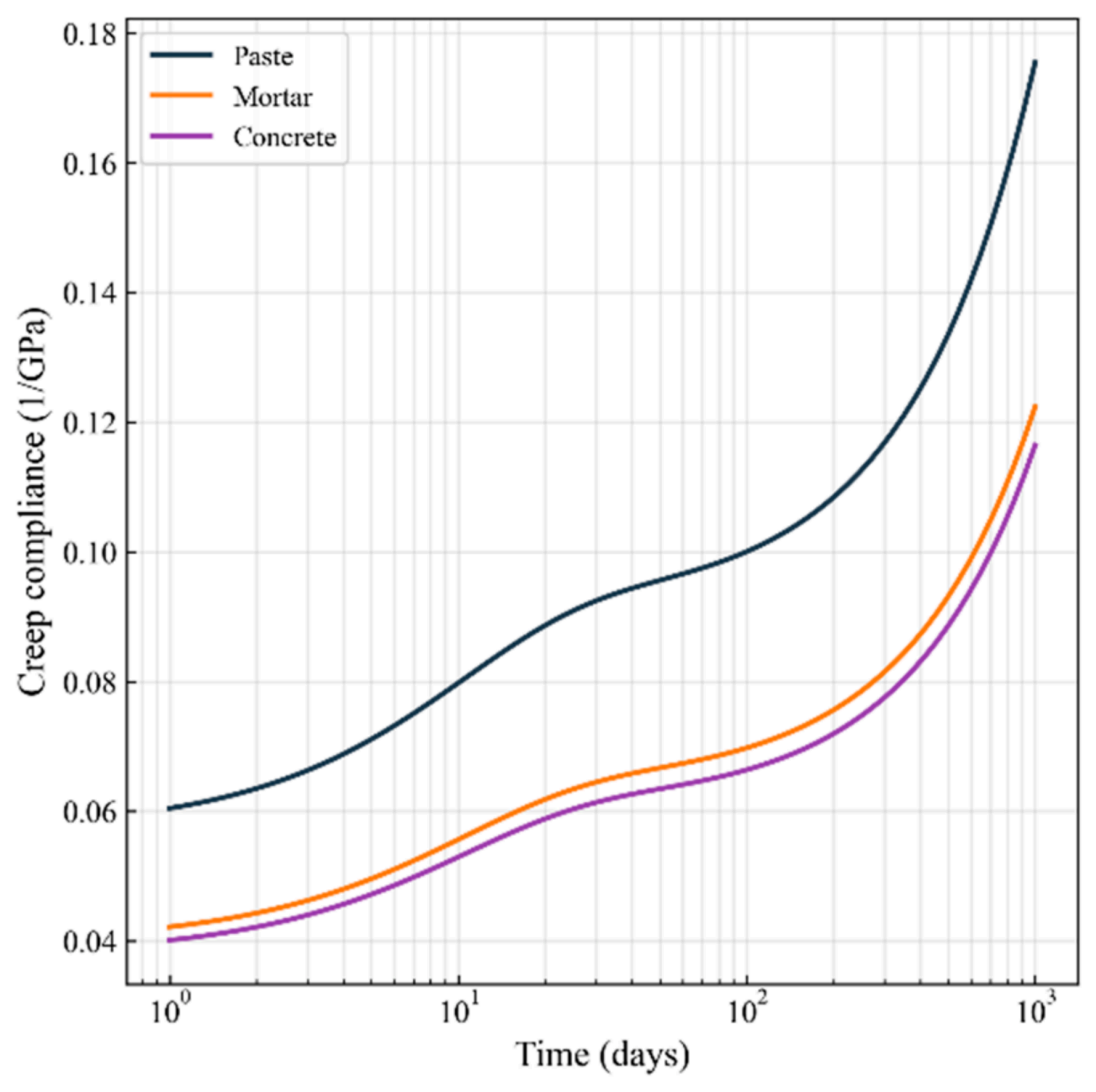
Comparison of creep compliance at different material scales.

The influence of material composition on creep compliance is examined through water–cement ratio, initial porosity, sand volume fraction, and material scale. The influence of water–cement ratio is shown in Figure 24. Three water–cement ratios are considered, namely 0.37, 0.47, and 0.57. A higher water–cement ratio leads to a more porous and less stiff microstructure, which is reflected in a lower elastic modulus. The predicted creep compliance increases with increasing water–cement ratio, and the difference becomes larger at later ages. At 365 days, the compliance for a water–cement ratio of 0.57 is about 12% higher than for 0.37. The physical explanation for this is that a higher porosity provides more space for the movement of water and for the rearrangement of C-S-H gel particles, promoting creep deformation. This result is in agreement with the general understanding that concrete with a higher water–cement ratio exhibits larger creep. A similar effect is observed for initial porosity, as shown in Figure 25. Three porosity values are considered, namely 0.15, 0.20, and 0.25. A higher initial porosity leads to a lower elastic modulus and a more open microstructure, which increases the creep compliance. The curves become more separated at later ages, since the viscous flow and delayed elastic deformation are both amplified in a more porous material. Physically, higher porosity provides more free space for the movement of water and for the rearrangement of C-S-H gel particles, which promotes time-dependent deformation. This result confirms that the initial porosity is a key factor in the long-term creep of concrete. The influence of sand volume fraction is illustrated in Figure 26. Three sand volume fractions are considered, namely 0.15, 0.231, and 0.30. A higher sand volume fraction reduces the creep compliance because the fine aggregates act as rigid inclusions that restrain the deformation of the surrounding cement paste. The effect is more significant at later ages, when the viscous flow dominates. This result is consistent with the general behavior of composite materials, where increasing the volume fraction of stiff inclusions lowers the overall creep. It also demonstrates that the multi-scale homogenization procedure can capture the stiffening effect of aggregates in a physically consistent manner. Finally, the effect of material scale is presented in Figure 27. The creep compliance is compared for cement paste, mortar, and concrete under the same corrosion condition. Cement paste has the highest compliance because it contains no stiff aggregates to restrain deformation. Mortar shows an intermediate compliance due to the presence of fine aggregates. Concrete exhibits the lowest compliance because both fine and coarse aggregates act as rigid inclusions that restrict the creep of the surrounding matrix. At 365 days, the compliance of cement paste is about twice that of concrete. This large difference demonstrates the crucial role of aggregates in limiting creep deformation and underlines the necessity of multi-scale modeling. These results confirm that both the initial pore structure and the aggregate volume fraction govern the long-term creep of concrete, and the multi-scale homogenization framework captures these effects consistently.

### 4.5. Limitations

The present model has several limitations that should be acknowledged.

(1) The link between the corrosion degree, the phase composition, and the creep behavior is phenomenological rather than directly validated. The phase–evolution relationships, via Equations (1)–(6), were calibrated using the QXRD and TG/DTG data of Jia et al. [3,5], and the Burgers creep parameters were calibrated using the creep data of Gong et al. [52]. However, direct QXRD/TG data for the creep specimens of Gong et al. [52] were not available. The model, therefore, assumes that the phase volume fractions are functions of the local CD and that the creep parameters depend on the corrosion product content, without direct physicochemical validation of this correlation. In addition, the model is calibrated and validated only over the CD range 0–0.5. Beyond CD = 0.5, the quadratic term in Equation (6) begins to dominate, and the coefficients may no longer represent the physical processes accurately. The mass/volume balance check in Table 4 is therefore limited to CD = 0–0.5. Future work should combine QXRD/TG/DTG analysis and creep testing on the same specimens to validate the phase–creep correlation directly, and the model should be extended to higher corrosion degrees with additional experimental data.

(2) The aggregates are assumed to be chemically inert and linear elastic under sulfate attack. This assumption is reasonable for the siliceous aggregates used in the referenced studies, but it may not hold for reactive aggregates such as limestone or recycled aggregates. For such materials, the chemical interaction between the aggregate and the sulfate solution may alter the ITZ properties, and the model would require recalibration of the ITZ phase–evolution relationships. The influence of aggregate type and mineralogical composition on ITZ degradation was not evaluated separately in the source studies. Similarly, the transfer of phase–evolution relationships between different material systems is based on the assumption that the local phase assemblage depends primarily on the local CD, regardless of the macroscopic material scale. This assumption is valid for OPC-based systems with comparable cement chemistry and aluminate contents. For different cement types, for very high sulfate concentrations, or for low-pH environments, the relationship between the external exposure and the local CD may change, and recalibration would be required. Recent studies on cement pastes exposed to aggressive solutions have shown that the chemical and mineralogical composition of the cement, particularly the content of aluminate phases, strongly influences the formation of ettringite and gypsum, along with the resulting degradation of the cementitious matrix [63,64,65]. Sujak et al. [63] and Durczak et al. [64] showed that the phase composition of Portland cement pastes changes significantly under aggressive solution exposure, and Pyzalski et al. [65] highlighted the role of C_3_A hydration in the formation of corrosion products. Although these studies focus on biological corrosion, their methodological approaches support the need to account for cement mineralogy and solution chemistry in sulfate-attack modeling. In the present model, these factors are reflected in the phase–evolution relationships and the corrosion degree mapping, but a systematic study of the effect of C_3_A content on the model parameters remains for future work. The applicability of the model is therefore restricted to OPC-based concrete with siliceous aggregates under the exposure conditions summarized in Table 5, and extension to other material systems requires combined chemical and mechanical characterization of the ITZ, as well as recalibration.

(3) The Burgers creep model captures the basic features of instantaneous, delayed, and viscous deformation, but it does not account for aging effects within the creep formulation itself. Aging is introduced only through the dependence of the elastic modulus on the loading age, which is a simplification. In reality, the creep compliance of concrete depends on the age at loading in a more complex way, and the aging effect can be significant for young concrete. A more physically based creep model, such as the solidification theory or the microprestress–solidification theory, could provide a more accurate representation of aging and drying effects. However, these models require additional parameters that are difficult to calibrate for sulfate-attacked concrete. In addition, the damage parameters α and η are treated as constants that depend only on the corrosion degree, but their values may also depend on the stress level and the exposure history. Under high stress, the microcracking induced by corrosion may interact with load-induced cracking, leading to a more rapid degradation. The present model does not account for this coupling. Extending the framework to include stress-dependent damage evolution would improve the predictive capability for structural applications.

(4) The spatial gradient of corrosion degree is represented by an exponential decay with a single decay coefficient β. The value of β is calibrated from the sulfate concentration profiles of Cheng et al. [12] as β ≈ 0.30 mm^−1^. With h_ITZ = 0.03 mm, the product β·h_ITZ = 0.009 ≪ 1, indicating that the corrosion degree is essentially uniform within the ITZ thickness under the exposure conditions considered here. This representation assumes that the sulfate concentration profile can be well approximated by an exponential function. In practice, the concentration profile may deviate from the exponential values due to chemical reactions, precipitation, and changes in transport properties over time. A more detailed description of the sulfate concentration profile could be obtained from numerical transport-reaction models, and the resulting profile could be directly used as input to the multi-scale framework. More importantly, the independent validation against the work of Piasta and Schneider [55] reveals a limitation related to stress effects. Under low compressive stress (σ/R = 0.35), the sulfate-induced expansion is restrained by the applied load, causing the time-dependent deformation to saturate rather than to grow linearly with time. The Burgers model contains a linear steady-state term and therefore, cannot reproduce this saturation; the predicted compliance continues to grow and overestimates the experimental values (MAPE = 42.35%). At moderate stress (σ/R = 0.50), the restraint effect is weaker, and the model performs well within the 0–540 day window (MAPE = 12.77%). This limitation restricts the applicability of the present framework to moderate stress levels and moderate exposure durations, for which the sulfate-induced deformation does not dominate the creep response. For long-term predictions under strongly restrained expansion, a stress-dependent or damage-coupled creep formulation would be required.

(5) The value CD = 0.3 assigned to acid rain in Section 4.3 is a scenario assumption, not a measured value. It is used only to illustrate the effect of environmental severity. The qualitative statement that acid rain is more aggressive than sulfate attack alone is supported by Peng et al. [59] and Yuan et al. [60], but no direct measurement of the equivalent corrosion degree under acid rain is available. The mapping from environment type to CD requires further experimental calibration.

Despite these limitations, the proposed model provides a useful engineering tool for predicting the long-term deformation of sulfate-attacked OPC concrete under moderate stress levels and moderate exposure durations. It captures the essential coupling between corrosion-induced phase changes and creep behavior, and it offers a clear path for future refinements through the incorporation of more accurate material parameters and environmental conditions.

## 5. Conclusions

The present study developed a corrosion-layered multi-scale homogenization framework for predicting the creep compliance of sulfate-attacked concrete. The framework couples phase evolution, ITZ corrosion layering, and viscoelastic creep. The phase evolution model was established from experimental measurements, expressing the volume fractions of major phases as functions of corrosion degree. The ITZ was discretized into sub-layers with exponentially decaying corrosion degree, allowing the spatial gradient of damage to be captured. The Mori–Tanaka homogenization scheme was integrated with a Burgers creep model, where the instantaneous elastic modulus is obtained from the multi-scale elastic calculation, and the creep parameters are modified by the corrosion product content. This framework was validated against literature data and was able to reproduce the non-monotonic evolution of elastic modulus and the increase in creep compliance with sulfate concentration.

(1) The Mori–Tanaka homogenization scheme successfully reproduces the non-monotonic evolution of elastic modulus under sulfate attack, with predictions within the experimental scatter. The phase evolution model captures the competitive processes of pore filling and microcracking.

(2) The Burgers creep model, coupled with the homogenized elastic modulus, provides reasonable predictions of creep compliance for sulfate-attacked concrete. The compliance increases with corrosion degree, reflecting the combined effects of stiffness reduction and increased long-term deformation.

(3) Sensitivity analyses reveal that the long-term creep modulus and the corrosion product coefficient are the most influential parameters controlling creep compliance. The loading age and wet–dry cycles also affect creep through their influence on the effective corrosion degree.

(4) The layered ITZ representation enables the model to capture the spatial gradient of corrosion, which is not possible with uniform corrosion models. This feature is essential for the accurate prediction of long-term deformation in structures with finite corrosion-affected thickness.

The proposed framework provides a basis for the service-life assessment of concrete structures in sulfate environments. Future work should focus on obtaining more accurate viscoelastic parameters for individual corrosion products and on extending the model to account for aging and multi-axial stress states.

## Figures and Tables

**Table 3:** Concrete mix proportions and raw material characteristics.

Item	Parameter	Value
Cement	Type	P.O 42.5 ordinary Portland cement
	Chemical composition (wt%)	SiO_2_ 26.01, Al_2_O_3_ 4.99, Fe_2_O_3_ 2.49, CaO 65.78, SO_3_ 0.55, Na_2_O 0.46, MgO 0.86, K_2_O 0.48
	Mineralogical composition (wt%)	C_3_S 31.5, C_2_S 50.8, C_3_A 9.0, C_4_AF 7.6
Aggregate	Fine	River sand, fineness modulus 2.7, 2570 kg/m^3^
	Coarse	Crushed siliceous gravel, 5–25 mm
Mix proportion	w/b	0.47
	Cement/Fly Ash/Water (kg/m^3^)	260/80/166
	Sand/Coarse Aggregate (kg/m^3^)	815/1040
	Water reducer (%)	2.8
Specimen	Type	100 × 100 × 400 mm prism
	Curing	28 d, 20 °C, RH ≥ 95%

**Table 4:** Mass/volume balance of the phase–evolution equations over CD = 0–0.5.

CD	*f_AFt_*	*f_gypsum_*	*f_CH_*	*f_LD_*	*f_HD_*	*f_pore_*	Raw Sum	Norm. Factor	Norm. Sum
0.0	0.0706	0.0000	0.1035	0.3286	0.2149	0.2824	1.0000	1.0000	1.0000
0.1	0.1006	0.0250	0.0835	0.2986	0.2099	0.2739	0.9915	1.0086	1.0000
0.2	0.1306	0.0500	0.0635	0.2686	0.2049	0.2684	0.9860	1.0142	1.0000
0.3	0.1606	0.0750	0.0435	0.2386	0.1999	0.2659	0.9835	1.0168	1.0000
0.4	0.1906	0.1000	0.0235	0.2086	0.1949	0.2664	0.9840	1.0163	1.0000
0.5	0.2206	0.1250	0.0035	0.1786	0.1899	0.2699	0.9875	1.0127	1.0000

## Data Availability

The original contributions presented in this study are included in the article. Further inquiries can be directed to the corresponding author.
